# Multi-fractal characterization of bacterial swimming dynamics: a case study on real and simulated *Serratia marcescens*

**DOI:** 10.1098/rspa.2017.0154

**Published:** 2017-07-12

**Authors:** Hana Koorehdavoudi, Paul Bogdan, Guopeng Wei, Radu Marculescu, Jiang Zhuang, Rika Wright Carlsen, Metin Sitti

**Affiliations:** 1 Department of Aerospace and Mechanical Engineering, University of Southern California, Los Angeles, CA 90089-1453, USA; 2 Department of Electrical Engineering, University of Southern California, Los Angeles, CA 90089-2560, USA; 3 Department of Electrical and Computer Engineering, Carnegie Mellon University, Pittsburgh, PA 15213, USA; 4 Department of Mechanical Engineering, Carnegie Mellon University, Pittsburgh, PA 15213, USA; 5 Department of Engineering, Robert Morris University, Pittsburgh, PA 15108, USA; 6 Physical Intelligence Department, Max-Planck Institute for Intelligent Systems, 70569 Stuttgart, Germany

**Keywords:** non-ergodicity, nonlinear dynamics, multi-fractality, bacterial motion, bacterial swimming

## Abstract

To add to the current state of knowledge about bacterial swimming dynamics, in this paper, we study the fractal swimming dynamics of populations of *Serratia marcescens* bacteria both *in vitro* and *in silico*, while accounting for realistic conditions like volume exclusion, chemical interactions, obstacles and distribution of chemoattractant in the environment. While previous research has shown that bacterial motion is non-ergodic, we demonstrate that, besides the non-ergodicity, the bacterial swimming dynamics is multi-fractal in nature. Finally, we demonstrate that the multi-fractal characteristic of bacterial dynamics is strongly affected by bacterial density and chemoattractant concentration.

## 1. Introduction

The future potential of using bacteria for therapeutic purposes [1,2] and regenerative medicine makes the dynamics of such microswimmers highly attractive to study. As an example, to overcome the penetration limitation of cancer chemotherapy, Toley & Forbes suggest using motile bacteria which can migrate into solid tumours as carrier of the drug in chemotherapy [3]. In another example, Felfoul and his co-workers show that magnetotactic bacteria can be used to transport drug-loaded nanoliposomes into the oxygen-depleted hypoxic region of the tumour [4]. Considering such applications, significant research efforts have been focused on analysing and modelling bacterial swimming dynamics. Broadly speaking, the mathematical models used to describe the bacterial swimming dynamics can be classified into two categories. The first category is based on a *microscopic* (i.e. cell-level) view of bacterial swimming through a set of equations where each equation describes the state of a single agent [5–9]. The second category provides a *macroscopic* (i.e. population-level) view via continuum-based partial differential equations that capture the dynamics of population density over space and time, without considering the intracellular characteristics directly [10–18]. Among the present models, Schnitzer [19] uses the Smoluchowski equation to describe the biased random walk of the bacteria during chemotaxis to search for food. To focus on a detailed description of the motion taking place during one run interval of the bacteria, de Gennes [20] derives the average run length travelled by bacteria during one counterclockwise interval. Along the same direction, to consider the environmental condition affecting the biased random walk of bacteria, Croze and his co-workers [21] study experimentally and theoretically the effect of concentration of soft agar on chemotaxis of bacteria. To study the effect of obstacles (another environmental condition) on the motion of bacteria, Chepizhko and his co-workers study the motion of self-propelled particles in a heterogeneous two-dimensional environment and show that the mean square displacement of particles is dependent on the density of obstacles and the particle turning speed [22,23]. Building on these models, Cates [24] highlights that bacterial dynamics does not always obey detailed balance, which means it is a biased diffusion process depending on the environmental conditions. Moreover, Ariel and his co-workers focus on diffusion of bacteria and show that the bacteria perform super-diffusion during swarming on a surface [25]. To add to the current knowledge, in this paper, we take into account realistic conditions like volume exclusion (i.e. no two bacteria can occupy the same space at the same time), chemical interactions among bacteria (i.e. autochemotaxis happening when bacteria excrete the converted substrate succinate molecules in the environment into chemoattractant aspartate molecules [26]), obstacles (e.g. during the drug delivery task, the existing cells and biological residues act as obstacles and interfere with the swimming bacteria) and heterogeneous distribution of chemoattractant in the environment and identify fractal characteristics of single bacterium motion, which could have a fundamental impact on mathematical modelling of both single bacterium and population swimming dynamics.

**Figure 1.**
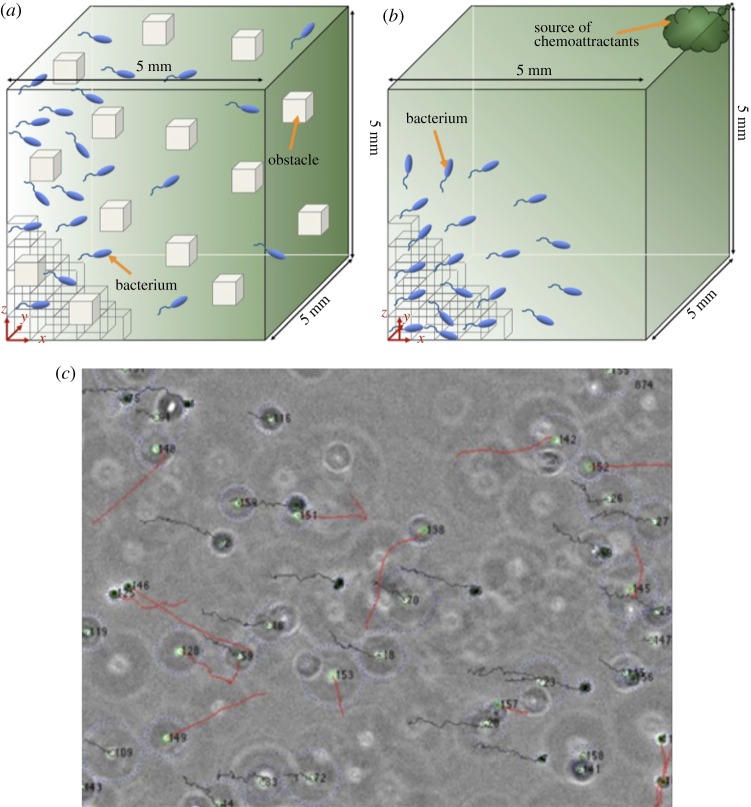
Simulation configuration of *S. marcescens* in BNSim (see §2) and single particle tracking of *S. marcescens* during experiments. (*a*) In case 1 for simulation in BNSim, a cubic environment with dimensions 5000 × 5000 × 5000 µm^3^ has been considered. The whole environment subdivided into 10^6^ smaller cubes, each with a size of 50 × 50 × 50 µm^3^. We consider a linear gradient (10^−4^ mM µm^−1^) of chemoattractant (l-aspartate) only in the *y*-direction and no gradient in other directions. (*b*) In case 2 for simulation in BNSim, we consider the same configuration as in case 1 for bacteria and the environment. We only changed the gradient of chemoattractant in the environment. In this case, we consider a linear gradient of chemoattractant (l-aspartate) from the injection location to the targets, meaning that there is a linear gradient in 3 different dimensions. (*c*) Single particle tracking of *S. marcescens* in an *in vitro* environment with dimensions of 10000 × 500 × 150 µm^3^ with linear gradient (10^−4^ mM µm^−1^) of chemoattractant (l-aspartate) in the second direction (*y*-direction).

We analyse the motion trajectories of *Serratia marcescens* [27,28]. Extracting single bacterium trajectories of motion from computer simulations for *S. marcescens* (figure 1*a*,*b* shows the different simulation set-ups that we used for the chemoattractant gradient in the environment) and applying single-cell visual tracking methods to real experiments on *S. marcescens* (figure 1*c*) reveals the fractal characteristics of bacterial motion.

To elucidate the complexity of the bacterial dynamics, we investigate the individual trajectories of simulated *S. marcescens* beside trajectories of real *S. marcescens* at multiple scales in space and time; this way, we show single bacterium motion has a phase transition from a super-diffusive behaviour to a normal diffusion and lastly to a sub-diffusive pattern based on bacterial density and chemoattractant distribution. We also show that bacterial motion exhibits a *multi-fractal* behaviour. The multi-fractal feature means that bacterial motion is self-similar, which is a concept dating back to the pioneering work of Kolmogorov to explain the chaotic behaviour [29,30]. Consequently, the multi-fractal formalism offers mathematical techniques to capture this nonlinear self-similarity in their motion.

The remainder of this paper is organized as follows. In the first section of results, we investigate the swimming dynamics of simulated *S. marcescens*. We explore the second-order moment of motion using an ergodicity test under different conditions including bacterial density and chemoattractant distribution. Then, we explore the third-order moment of motion with a nonlinearity test. Next, we discuss that focusing on second- and third-order moments of a single bacterium's motion cannot capture the entire spatial and temporal complexity of the motion; therefore, we perform multi-fractal analysis which considers higher-order moments of their motion. We show that the simulated *S. marcescens* bacterial swimming displays multi-fractal characteristics*.* In the last section of results, using experimental swimming trajectories of *S. marcescens*, we show that their motion is multi-fractal, validating our conclusions for simulated bacteria. Finally, the discussion section concludes the paper and outlines some future research directions.

## 2. Material and methods

### (a) Experiments

Laboratory experiments were conducted to measure the swimming behaviour of live and motile *S. marcescens* under two conditions: (i) an isotropic environment (i.e. uniform distribution of substances) with dimension equal to 10 000 × 500 × 150 µm^3^ and (ii) a stable linear gradient of l-aspartate, which is a canonical chemoattractant for bacterial species such as *S. marcescens*.

#### (i) Bacterial culture

To conduct the experiments, *S. marcescens* (ATCC 274, American Type Culture Collection, Manassas, VA, USA) was cultured using established protocols [28,31–33]. The bacteria were first grown to reach the exponential state in their growing process (during this state, the number of bacteria doubles at an approximately constant rate, which is around every 20 min for *S. marcescens*) in a liquid culture (25 g of Difco LB Miller Broth and 1 l of deionized (DI) water, pH 7.0) on a shaker at 37°C for 3.5–4 h. Then, an aliquot of 2.0 µl of the liquid culture was placed on an agar plate (25 g of Difco LB Miller Broth, 6 g of Bacto Agar, 5 g of glucose, 1 l deionized water), and the agar plate was incubated at 30°C for 16–20 h. For the experiments, bacteria from the leading edge of the colony were extracted and then diluted in motility buffer (0.01 M KH_2_PO_4_, 0.067 M NaCl, 0.1 mM EDTA, pH 7.0) to appropriate densities for the measurements. Subculturing the bacterial cells on an agar plate allows the most motile bacterial cells, which have been shown to be located along the leading edge of a spreading colony [28], to be selectively chosen for the study.

#### (ii) Experimental set-up

A three-channel microfluidic concentration gradient generator was fabricated to measure bacterial swimming parameters under chemotaxis and without chemotaxis; detailed descriptions of the device can be found in Zhuang *et al*. [27], and a figure of the device is shown in the electronic supplementary material, figure S10. The gradient generator consisted of three parallel microfluidic channels within an agarose gel. By controlling the concentration of the chemoattractant in the outer two channels of the device, a linear chemoattractant concentration profile could be generated by molecular diffusion in the middle channel in which the bacterial suspension was placed. The gradient generator was calibrated by observing the diffusion of 10^−4^ M fluorescein (Sigma-Aldrich Co.) in the device as described in Zhuang *et al*. [27] and Edwards *et al*. [28]. Fluorescence intensity profiles from these studies revealed that a stable linear gradient developed within 20 minutes. Given that the diffusion coefficient of l-aspartate is 9.0 × 10^−6^ cm^2^ s^−1^ and the diffusion coefficient of fluorescein is about half that of l-aspartate [27], it can be concluded that about 10 min are required to develop a stable chemoattractant concentration gradient of l-aspartate and the gradient remains stable beyond 10 min. In our study, the bacterial swimming motion was measured after 15 min, ensuring a stable linear concentration gradient within a quiescent fluid environment.

#### (iii) Experimental conditions

Chemotaxis of *S. marcescens* was measured under an l-aspartate gradient of 0.2 mM mm^−1^, with an average concentration of 0.1 mM. To measure the bacterial swimming behaviour in an isotropic environment, we used the same device but with a uniform distribution of motility buffer (without an l-aspartate gradient). For both cases, with and without the chemoattractant gradient, the bacterial swimming parameters were studied for three different cell densities, which were 10^6^ bacteria cm^−3^, 10^7^ bacteria cm^−3^ and 10^8^ bacteria cm^−3^. All the experiments were conducted at room temperature (19–22°C).

#### (iv) Imaging and tracking

The bacteria were imaged with an inverted microscope (40× objective, Zeiss AxioObserver.A1, Carl Zeiss, Oberkochen, Germany), and videos were obtained at 88 frames per second with a CCD camera (Foculuc FO134SB). All video data were acquired at least 20 µm (approx. 20 body lengths) from any surface to minimize the wall effects on the bacterial swimming behaviour, and no obstacles hindered the movement of the bacteria (except for other bacterial cells). The bacteria were tracked in three dimensions by an in-house visual tracking program developed in Matlab (R2012a, The MathWorks, Inc, Natick, MA, USA). The tracking program extracts the *x–y* positions of bacteria in the video frames using image thresholding techniques, while the *z*-position was obtained by making use of the lens aberrations created by out-of-focus bacterial cells. This defocused optical tracking method has been applied by several groups to track the three-dimensional motion of bacteria and small particles [28,34–37]. A linear relationship exists between the radius of the aberration ring appearing around an out-of-focus cell and the cell's vertical distance (along *z*-axis) to the focal plane. The following equation exhibits this relation:

2.1 $$r=c_{1}|z|+c_{0}.$$

In the above equation, *c*_0_ and *c*_1_ were calibrated by performing an experiment in which the bacteria were fixed a known distance away from the focal plane. The variables were determined from Edwards *et al*. [28]. Therefore, the *z*-position of a bacterial cell could be determined by measuring the size of the aberration ring around the cell (for more details see Note 12 in the electronic supplementary material).

### (b) Simulations

For completeness of our analysis, we used computer simulations of known mathematical models for the chemical pathways of *S. marcescens* to capture their dynamics through simulations in addition to experimental data. The main reason for using simulations beside real experiments is the ergodicity test. For performing the ergodicity test and measuring the time-averaged mean square displacement and the ensemble mean square displacement, we need at least 1000 trajectories of exactly the same bacteria starting with exactly the same initial condition. Owing to constraints on experimental conditions, we are not able to perform the same experiment 1000 times exactly with the same initial conditions to track exactly the same bacteria in all of them. To tackle this issue, we used BNSim [38], an open-source, parallel multi-scale stochastic modelling platform integrating various simulation algorithms with genetic circuits and a chemotaxis pathway modelled in a complex three-dimensional environment (for the detailed mathematical model of chemotaxis pathway, see Note 6 in the electronic supplementary material). Specifically, to simulate the chemoreceptors, we used the Monod–Wyman–Changeux model in which the receptor homodimers assemble into fully cooperative signalling teams that switch rapidly between active and inactive states [39]. The methylation kinetics is based on the celebrated Barkai and Leibler model for a near-perfect adaptation system [40,41]. For the signal transduction from chemoreceptor to the flagella motor regulator Yp, the concentration of phosphorylated CheYp is assumed to be proportional to the kinase activity without considering the nonlinear dependence [42]. Finally, we used a two-state model to describe the motor behaviour of bacteria, which sets the clockwise (CW) and counterclockwise (CCW) states in two potential wells [43,44]. The transition rates have been fitted to experimental data [27,28,45]. For the interactions among cells, we consider volume exclusion effects and also autochemotaxis (chemical interaction) between the bacteria inside the swarm in all of our simulations. Autochemotaxis happens when bacteria excrete the converted substrate succinate molecules in the environment into chemoattractant aspartate molecules [26,46–48]. Once the succinate gets depleted, bacteria start consuming the chemoattractant aspartate excreted by themselves and they may detect each other's presence through this process [24]. Of note, this form of chemical interaction has been defined as *cue* in the chapter entitled Communication in bacteria by Diggle and his co-workers in [49]. (Note 10 in electronic supplementary material explains how autochemotaxis has been considered in the computational model).

We performed simulation for *S. marcescens* with two different speeds considering three different cases as follows. For some cases we used a higher swimming speed that is more appropriate to *Escherichia coli* than to *S. marcescens* (simulation cases 1 and 2). The simulation of the chemical pathway is the same for all the cases.

The simulations have been done for three different cases.

#### (i) Case 1

We used a cubic environment with dimension 5000 × 5000 × 5000 µm^3^ (figure 1*a*). The whole environment was subdivided into 10^6^ smaller cubes, each with a size of 50 × 50 × 50 µm^3^. The rationale for using these small cubes was to capture bacterial interaction with nearby bacteria and the environment efficiently. The three-dimensional space is therefore tessellated in cubes, while a bacterium is a sphere with a radius of 1 µm. We also used these smaller cubes to model obstacles in our simulations. The way we implement the obstacles in our simulation is by making these small cubes impenetrable; thus, when a bacterium approaches one of these cubes, it cannot continue swimming and so it has to choose a new direction for runs. Simulated bacteria had a constant run speed of 14 µm s^−1^ and a rotational diffusion constant of 0.062 rad^2^ s [50,51]. After each tumble, cells were reoriented in a new direction that is randomly sampled from a gamma distribution with a scale parameter of 18.32 and a shape parameter of 4, matching the experimentally observed distribution of new run angle.

In this set of simulations, different population densities of 8 × 10^2^ bacteria cm^−3^, 8 × 10^3^ bacteria cm^−3^ and 8 × 10^4^ bacteria cm^−3^ in the environment were considered. These population densities correspond to a total number of 10^2^ bacteria, 10^3^ bacteria and 10^4^ bacteria, respectively. To study the effect of chemotaxis on the environment, we considered a steady-state linear gradient (10^−4 ^mM µm^−1^) of chemoattractant (aspartate) in the *y*-direction. We simulated each bacterial density considering two different conditions: with the gradient of chemoattractant in the environment and without the gradient of chemoattractant in the environment.

Besides population density of bacteria, existence of obstacles will affect bacterial motion. In realistic scenarios of using bacteria for drug delivery *in vivo*, the swarm dynamics will be affected by collisions with existing obstacles. For instance, during the drug delivery task, the existing cells and biological residues act as obstacles and interfere with the swimming bacteria. To analyse the effect of obstacles on bacterial motion, we considered 0.01% of the small cubes uniformly distributed in the environment to be impenetrable (i.e. totally 10^4^ number of cubes are impenetrable); thus, when the bacterium hits one of these cubes, it cannot continue to enter the cube and it will continue tumbling to choose a new direction for its run (Note 8 in electronic supplementary material explains how the obstacles have been applied to BNSim in more detail).

It is important to mention that the obstacles' effect on single bacterium motion is different from the impact of bacteria population density based on volume exclusion effects. Each bacterium tries to avoid collision with the obstacles around it, meaning that the obstacles affect single bacterium motion. On the other hand, bacteria population density also affects bacterium motion (directly and indirectly). This means each bacterium modifies its motion to preventing collision with other bacteria (directly). Moreover, the chemical interaction between the bacteria based on their population density will affect the motion of each bacterium (indirectly). In other words, autochemotaxis between the bacteria is dependent on bacteria population density. Therefore, it is important to investigate the effects of obstacles and bacterial density on single bacterium motion separately based on the differences in the nature of their effects.

#### (ii) Case 2

In this case, we had the same set-up for environment and simulated bacteria as for case 1. The only difference was in the population densities we simulated and the chemoattractant gradient in the environment.

In this case, we considered a wider range of population densities than in case 1, namely 8 × 10^2^ bacteria cm^−3^, 8 × 10^3^ bacteria cm^−3^, 8 × 10^4^ bacteria cm^−3^, 8 × 10^5^ bacteria cm^−3^ and 8 × 10^6^ bacteria cm^−3^ in the environment. These population densities correspond to the total number of bacteria, from 10^2^ bacteria up to 10^6^ bacteria in the simulations. The other significant difference between this case and case 1 was that we considered a steady-state radial gradient (10^−4^ mM µm^−1^) of chemoattractant (aspartate) from the injection location towards the targeted region, namely ((*x*^2^ + *y*^2^ + *z*^2^)^0.5^ × 10^−4^ mM µm^−1^). We simulated each bacterial density considering two different conditions: with the gradient of chemoattractant in the environment and without the gradient of chemoattractant in the environment.

#### (iii) Case 3

In this case of simulation, we used a different swimming speed for bacteria compared to that of cases 1 and 2. Moreover, we used a different dimension for the environments. We used a three-dimensional environment with dimensions 10 000 × 500 × 150 µm^3^. The whole environment was subdivided into smaller cubes each with a size of 50 × 50 × 50 µm^3^. For the simulation, densities of 10^4^ bacteria cm^−3^, 10^5^ bacteria cm^−3^, 10^6^ bacteria cm^−3^ and 10^7^ bacteria cm^−3^ in the environment were considered. These population densities correspond to total numbers of 7 bacteria, 75 bacteria, 750 bacteria and 7500 bacteria, respectively. To study the effect of chemotaxis in the environment, we considered a steady-state linear gradient (10^−4^ mM µm^−1^) of chemoattractant (aspartate) in the *y*-direction. We simulated each bacterial density considering two different conditions: with the gradient of chemoattractant in the environment and without the gradient of chemoattractant in the environment.

## 3. Results

### (a) Investigation of simulated *S. marcescens* swimming dynamics

#### (i) Exploring the second-order moment of motion with the ergodicity test

The dynamics of a bacterial population is affected by numerous factors, such as the spatio-temporal gradient of chemoattractant within the environment [52,53], bacteria density, volume exclusion [54], the intensity of autochemotaxis [55] and saturation of bacteria receptors. One important consequence of the mentioned dependencies is that the collective motion of bacteria is non-ergodic in nature [56–58]. To investigate this, we performed an ergodicity-breaking test which checks that long-time averages differ from ensemble averages of bacterium displacement. Simply speaking, if the dynamics of a bacterium's motion is non-ergodic, the time-averaged mean square displacement (TAMSD) (i.e. $\bar{\lim_{T\to \infty }\delta^{2}(\Delta =t,T)}=(1/T-\Delta )\int_{0}^{T-\Delta }{\left|r(t+\Delta )-r(t)\right|}^{2}\text{d}t$) is not equal to the ensemble mean square displacement (EMSD) (i.e. $\left\langle r^{2}(t)\rangle =\int r^{2}P(r,t){\text{d}}^{3}r=\langle |r(t)-r(0){|}^{2}\right\rangle$). In mathematical terms, the system is non-ergodic if $\bar{\lim_{T\to \infty }\delta^{2}(\Delta =t,T)}\neq \langle r^{2}(t)\rangle$ [59–61]. In the TAMSD formula, Δ and *T* represent the lag time and the overall measurement time, respectively. In the EMSD formula, *P*(*r*, *t*) is the probability density function to find bacteria at position *r* at time *t* (in the TAMSD formula, the overline denotes the time average). It is important to mention that *in silico* simulation of bacteria provides the opportunity of performing the same simulation with exactly the same initial condition over 1000 times and of monitoring the behaviour of an identical bacterium in all simulations. This helps us to calculate the ensemble mean square displacement of a bacterium and to investigate the ergodicity test. Such analysis cannot be done for experiments *in vitro,* which is an advantage of *in silico* simulations to study the dynamics of bacteria.

**Figure 2.**
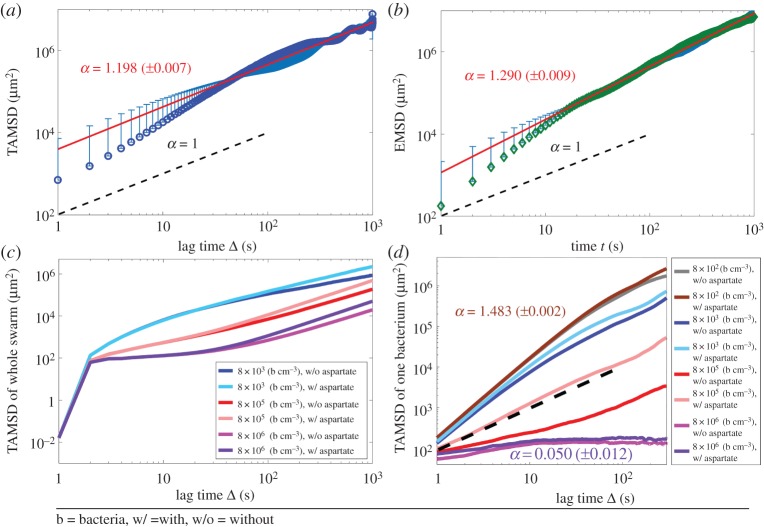
Mean square displacement (MSD) plots for simulated *S. marcescens* in BNSim. (*a*) Time-averaged MSD of one *S. marcescens* bacterium motion simulated in BNSim as a function of lag time $\Delta (\bar{\delta^{2}(\Delta ,T)}=(1/T-\Delta )\int_{0}^{T-\Delta }|r(t+\Delta )-r(t){|}^{2}\text{d}t)$ for a density of 8 × 10^4^ (bacteria cm^−3^) in an environment with a linear gradient of chemoattractant and without obstacles. This plot shows super-diffusion behaviour $\left(\text{MSD( }t\text{) }=k_{\alpha }t^{\alpha },\;1<\alpha <2\right)$ (configuration case 1 in §2). (*b*) Ensemble average MSD of *S. marcescens* motion simulated in BNSim versus time *t* calculated from the formula ($\text{MSD( }t\text{) }=\langle |r(t)-r(0){|}^{2}\rangle$) over 100 trajectories for a density of 8 × 10^4^ (bacteria cm^−3^) with a linear gradient of chemoattractant and without obstacles. This plot also shows super-diffusion behaviour. The observed difference between these two plots (*a*,*b*) shows an ergodicity-breaking behaviour in the system and demonstrates the non-ergodicity of bacterial dynamics (configuration case 1 in §2). (*c*) The TAMSD of the centre of the whole population for *S. marcescens* motion simulated in BNSim shows the effect of population density and chemoattractant on the centre of the bacteria population motion (configuration case 2). (*d*) The TAMSD of one *S. marcescens* trajectory simulated in BNSim for different cases shows the effect of chemoattractant and density on single bacterium motion (configuration case 2 in §2). By increasing the population density, we observe a phase transient in bacterial behaviour from super-diffusion to normal diffusion and then sub-diffusion (configuration case 2 in §2).

Figure 2*a–d* summarizes the ergodicity investigations on the simulated bacterial swimming dynamics under various bacterial densities, chemoattractant gradient and autochemotaxis effects. For instance, figure 2*a*,*b* show the difference between TAMSD and EMSD plots for a simulated bacterium in a population of 8 × 10^4^ bacteria cm^−3^ in an environment with a linear gradient of chemoattractant and without obstacles except other bacteria. More precisely, the TAMSD for this case when the lag time *t* = 10 (s) is *δ*^2^(*t* = 10, *T* = 1000) = 15 631 (µm^2^), while the EMSD obtained over 100 trajectories for the same timing conditions is $\left\langle r^{2}(t=10(\text{s}),T=1000(\text{s}))\rangle =12961\;(\mu m^{2}\right)$. The difference between the TAMSD and the EMSD in this case is 20%. The TAMSD for a lag time *t* = 10 s is independent of the initial time reference, which means that bacteria will move a distance equal to 125.02 (µm) on average. In contrast, the EMSD for the same time *t* *=* 10 (s) implies that bacteria will travel (on average) a distance of 113.84 (µm), which has a 10% difference. The significant difference between the TAMSD and the EMSD shows that, for simulated bacteria, the motion is non-ergodic and time-dependent [58]. Comparing the slopes of the lines fitted to both TAMSD and EMSD plots confirms that the single bacterium motion is non-ergodic (the slope of the line fitted to the TAMSD plot is 1.198 (±0.007), while that for the EMSD plot is equal to 1.290 (±0.009)). For completeness, we test all cases for different points in time (electronic supplementary material, table S2). The cases we analysed cover different bacterial densities and chemoattractant distribution in the environment. In all of these cases, the equality between the TAMSD and the EMSD does not hold; this confirms the non-ergodicity of the bacterial motion. In conclusion, the non-ergodicity finding holds for both case studies considered: (i) the effect of bacterial density and (ii) the effect of chemoattractant distribution in the environment.

The non-ergodicity of single bacterium motion follows anomalous diffusion behaviour based on EMSD plots. Generally speaking, the anomalous diffusion is a diffusion process characterized by a nonlinear relationship of the EMSD with time [62–66]. In contrast, a normal diffusion process has an EMSD that varies linearly with time. Figure 2*b* shows bacteria-driven motion displays a *super-diffusion* type of behaviour [67], which can be modelled using a power law form of the second moment with exponent ranging between 1 and 2 $\left(\text{i.e. }\bar{\left\langle \delta^{2}(\Delta ,T)\right\rangle }=K_{\alpha }\Delta^{\alpha },1\leq \alpha \leq 2\right)$ for different bacterial density cases. The *super-diffusive* behaviour of non-ergodic bacterium motion cannot be modelled with a Brownian random walk with an average drift which has ergodic characteristics.

**Figure 3.**
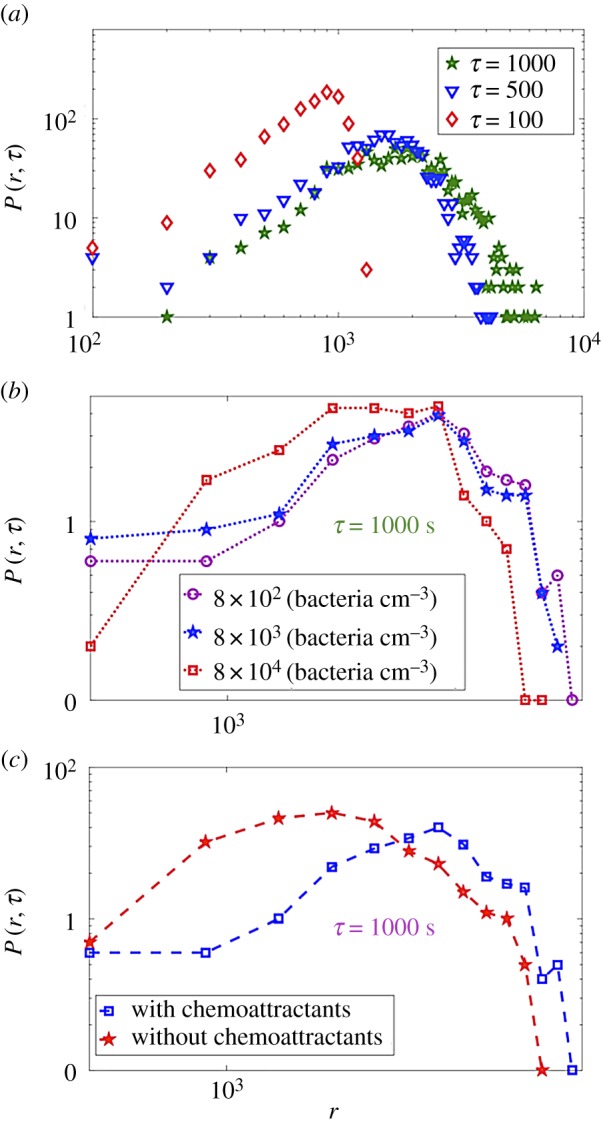
Effects of time, bacteria population density and chemoattractant on *S. marcescens* motion simulated in BNSim. (*a*) Effect of time on bacterial motion. Log–log plot of *P*(*r*, *τ*) as a function of distance *r* for different lag times *τ* = 100 s, 500 s, 1000 s. This result is for 8 × 10^2^ bacteria cm^−3^ in an environment without chemoattractant and obstacles. This plot shows that the bacteria move more with time and get further away from their initial locations. The peak of the plot decreases more (configuration case 1 in §2). This plot exhibits a stable type of distribution (electronic supplementary material, table S4). (*b*) Effect of density on bacterial motion. Log–log plot of *P*(*r*, *τ*) for lag time *τ* = 1000 s compared for different bacterial densities simulated in BNSim in an environment with chemoattractant gradients and without obstacles. By increasing the bacterial population, their motion will be restricted and they are able to move less freely in the environment (configuration case 1 in §2). (*c*) Effect of chemoattractant on bacterial motion. Log–log plot of *P*(*r*, *τ*) for lag time *τ* = 1000 s compared for the case with and without chemoattractant in the environment for the population of 8 × 10^2^ bacteria cm^−3^. By adding chemoattractant in the environment, the bacteria tend to move more and oscillate less, so they get further away from their initial conditions in the direction of increasing gradient of chemoattractant (configuration case 1 in §2).

Figure 2*c*,*d* shows that the single bacterium trajectory and the movement of the centre of the group exhibit super-diffusion behaviour for some cases in our simulations. The super-diffusion behaviour predicts that its corresponding random walk is characterized by a stable distribution [68,69]. To further elucidate this behaviour of the simulated bacteria, we investigate the histogram of the distance travelled by bacteria *P*(*r*, *τ*) in a specific lag time *τ* (figure 3*a*). It can be observed that it exhibits a stable type of distribution (electronic supplementary material, table S4). The origin of this behaviour is the lack of nutrient concentration in our simulation model, which leads to a high level of noise in the methylation model. This noisy fluctuation in the methylation model is the reason for the power-law run-length distribution in simulated bacterium motion [68]. Figure 3*a* shows that the side peak of the bacterium displacement histogram *P*(*r*, *τ*) shifts towards the right-hand side and broadens with time. Based on the Generalized Central Limit Theorem, the histogram will converge to a stable distribution as the lag time *τ* increases [70,71].

Figure 2*c*,*d* shows the effect of population density on the TAMSD for different bacterial density cases. The TAMSD variation of the centre of the entire bacterial population as a function of time lag Δ (figure 2*c*) shows that increasing the bacterial density makes the population less motile. Volume exclusion effects and chemical interactions caused by autochemotaxis between bacteria are the main reasons of this behaviour (Note 7 in electronic supplementary material explains how volume exclusion effects have been considered in the computational model).

Figure 2*d* shows the TAMSD of a single bacterium, which is in agreement with the results obtained from the TAMSD of the entire swimming trajectory. One reason for this behaviour is that increasing their population causes a significant increase in the autochemotaxis (Note 10 in electronic supplementary material explains how autochemotaxis has been considered in the computational model). In turn, this may cause receptor saturation which can ‘confuse’ bacteria in choosing its preferred direction of motion in the environment; this means that their motion may become restricted due to their high accumulation in the environment. Hence, the TAMSD of bacterial motion decreases (for the same time interval) with the increase in bacterial density.

Figure 2*d* magnifies the volume exclusion effect for the case of 8 × 10^6^ bacteria cm^−3^ (purple and magenta lines). As one can see, in this case, the bacterium is not able to move and therefore it remains in the neighbourhood of its original location. By increasing the bacterial population up to 8 × 10^5^ bacteria cm^−3^, we observe a phase transition in their motion from a super-diffusive behaviour to a normal diffusion and lastly to a sub-diffusive pattern (electronic supplementary material, table S3).

Besides the TAMSD plots, the displacement histogram *P*(*r*, *τ*) shows a higher probability for a longer displacement in the same time interval related to lower bacterial densities (figure 3*b*). When constraints like other bacteria in the environment limit a bacterium's motion, then the bacterium tends to oscillate more and randomly move in the environment rather than moving directionally.

We also study the effect of l-aspartate gradient on the TAMSD of bacterial motion trajectory. Figure 2*c*,*d* shows that by adding l-aspartate gradient (configuration case 2 in §2) to the environment, independent of bacterial density, the TAMSD will increase for both a single bacterium and an entire population for simulated bacteria. Moreover, figure 3*c* presents the histogram of displacement of bacteria after lag time *τ* (i.e. *P*(*r*, *τ*)) for the case with/without l-aspartate gradient in the environment. This figure shows that the probability of covering longer displacements (after the same time lag) by bacteria is higher in the case of l-aspartate gradient in the environment, meaning that bacteria tend to move directionally along a targeted direction and oscillate less when they sense a chemoattractant gradient in their environment while their instantaneous speed stays the same (figure 3*c*).

Up to this point, we focus on the non-ergodicity of single bacterium motion by investigating the second-order moment of their motion under different environmental condition. In what follows, we shift our focus to the third-order moment of their motion by performing a nonlinearity test.

#### (ii) Exploring the third-order moment of motion with nonlinearity test

Details about the first and second moments of the bacterial trajectory (corresponding to mean square displacement of bacterial motion) do not provide enough information regarding the complexity of their dynamics. Consequently, we analyse the higher-order moments of a bacterium's trajectory by investigating the skewness metric. Skewness represents the third-order moment of bacterium motion and helps to investigate whether or not the motion increments (i.e. changes in the spatial position of the bacterium in three-dimensional space between two different time instances) obtained from the bacterium trajectory obey a multivariable normal distribution in the three-dimensional environment. Non-zero skewness shows that the probability density function of motion increment does not follow the normal distribution and this is a signature of nonlinearity [72].

Using the Mardia method [72–74], we measured the multivariate skewness of our data for simulated bacteria (see electronic supplementary material, equation (2)). The non-zero highly variable skewness plots demonstrate that bacterial motion cannot be modelled by a multivariable normal distribution for all the cases under consideration (i.e. with and without chemoattractant and obstacles in the environment); this confirms the nonlinear behaviour (skewness plot in electronic supplementary material, figure S1).

To provide a more comprehensive analysis on the existence of nonlinear behaviour, we employed several statistical methods like Henze–Zirkler's multivariate normality test [75–77], Royston's multivariate normality test [78–80] and Doornik–Hansen omnibus multivariate normality test [81,82]. Electronic supplementary material, table S1 summarizes the results for these tests and further demonstrates the nonlinearity of simulated bacterium dynamics.

#### (iii) Exploring higher-order moment of motion using multi-fractal analysis

Traditional analysis of bacterial motion has focused on measuring the root mean squared fluctuations of the displacement and thus is limited to first- and second-order moment analysis. However, relying on the second-order statistics not only is inconsistent with the observed non-Gaussian behaviour, but also it does not capture the entire spatial and temporal complexity structure of the interactions among bacteria and the environment. Consequently, we employ concepts from statistics (e.g. higher-order moments) and fractal theory (e.g. multi-fractal spectrum, generalized Hurst exponent) to perform a multi-fractal [83,84] analysis and quantify the correlation structure and complexity of bacterium trajectories (see Note 5 in electronic supplementary material for more details). More precisely, we compute the generalized Hurst exponent as a function of the higher-order moment *q* and the multi-fractal spectrum. The generalized Hurst exponent of a bacterium trajectory measures the scaling and memory properties of the *q*th order moment of the distribution of fluctuations. For example, the generalized Hurst exponent for *q* = 2 coincides with the autocorrelation function. In addition, the generalized Hurst exponent helps us distinguish between mono-fractal and multi-fractal behaviour. If the generalized Hurst exponent proves to be independent of the order *q* of the moments, then the bacterium motion can be classified as mono-fractal. In contrast, if the generalized Hurst exponent exhibits any dependence on order *q*, then the bacterium motion is regarded as multi-fractal. Alternatively, we can investigate the existence of a multi-fractal behaviour by analysing the wideness of the multi-fractal spectrum (i.e. *W* = *h*(*q*)_max_ − *h*(*q*)_min_ estimating the range of present Holder exponents in the data). A wider multi-fractal spectrum shows that the multi-fractality degree is stronger, meaning that the motion structure is more complex and inhomogeneous. Therefore, a wider range of fractal exponents are needed to cover the complex structure of the bacterium motion. Another measure for multi-fractality degree is the most probable Holder exponent *h*(*q*) corresponding to the peak of the multi-fractal spectrum (i.e. *h*_0_(*q*)). Low *h*_0_(*q*) shows that the motion of bacteria is more correlated and regular in appearance, meaning that the motion is mono-fractal. Overall, multi-fractal analysis is an applicable tool to characterize the variability and heterogeneity in bacterium motion structure [85,86].

**Figure 4.**
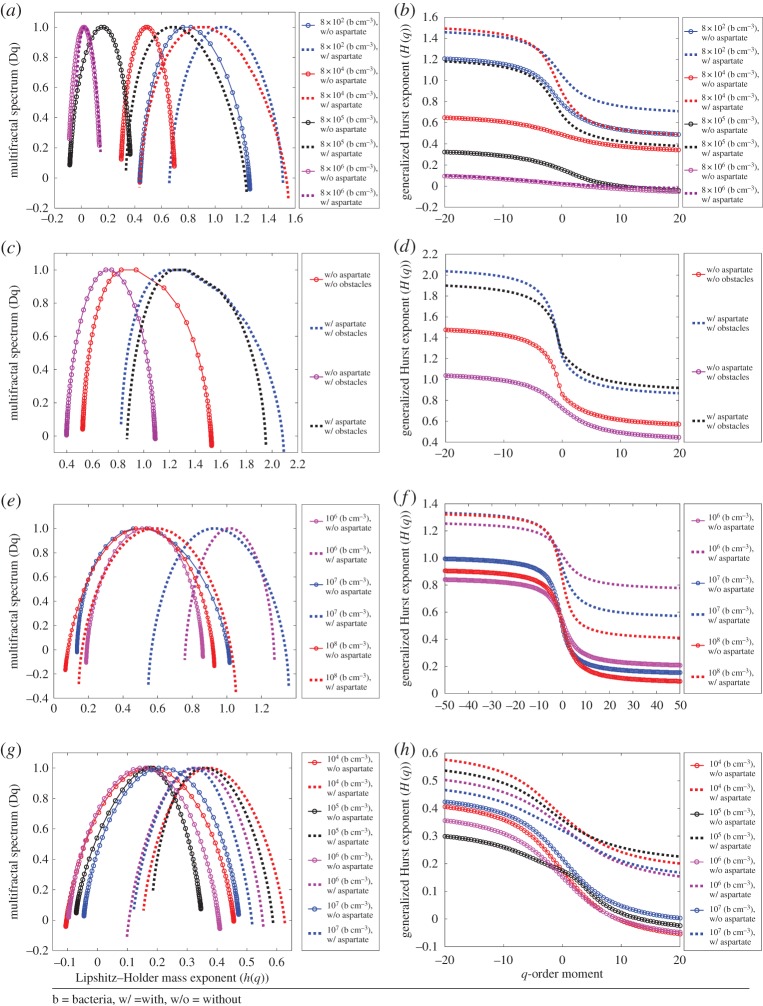
Multi-fractal analysis of motion for simulated *S. marcescens* in BNSim and *S. marcescens in vitro* experiments. (*a*) Multi-fractal spectrum of the simulated *S. marcescens* position in the direction of the linear gradient of chemoattractant for different cases with and without chemoattractant and different bacterial densities (configuration case 2 in §2). (*b*) Generalized Hurst exponent of the simulated *S. marcescens* position in the direction of the linear gradient of chemoattractant for different cases with and without chemoattractant and different bacterial densities (configuration case 2 in §2). (*c*) Multi-fractal spectrum of the simulated *S. marcescens* position in the direction of the linear gradient of chemoattractant with a density of 8 × 10^4^ bacteria cm^−3^ for different cases with and without chemoattractant and obstacles in the environment (configuration case 1 in §2). (*d*) Generalized Hurst exponent of the simulated *S. marcescens* position in the direction of the linear gradient of chemoattractant with a density of 8 × 10^4^ bacteria cm^−3^ for different cases with and without chemoattractant and obstacles in the environment (configuration case 1 in §2). (*e*) Multi-fractal spectrum of the experiments *in vitro* on *S. marcescens* position in the direction of the linear gradient of chemoattractant for different cases with and without chemoattractant and different bacterial densities. (*f*) Generalized Hurst exponent of the experiments *in vitro* on *S. marcescens* position in the direction of the linear gradient of chemoattractant for different cases with and without chemoattractant and different bacterial densities. (*g*) Multi-fractal spectrum of simulated *S. marcescens* position in the direction of the linear gradient of chemoattractant for different cases with and without chemoattractant and different bacterial densities (configuration case 3 in §2). (*h*) Generalized Hurst exponent of simulated *S. marcescens* position in the direction of the linear gradient of chemoattractant for different cases with and without chemoattractant and different bacterial densities (configuration case 3 in §2).

Figure 4*a–d* presents our results from the multi-fractal detrended fluctuation analysis [87,88] on motion trajectories of simulated *S. marcescens* in BNSim for different bacterial densities with and without the l-aspartate gradient in the environment (see simulation section in §2). Based on the simulation results (figure 4*a*), we observe that increase in the density of bacteria decreases the width of the multi-fractal spectrum (*W*) and decreases (*h*_0_(*q*)) as two different measures of the degree of multi-fractality. For instance, in the case of chemoattractant in the environment, the wideness (*W*) of the multi-fractal spectrum is shrinking from *W* = 0.851 to *W* = 0.182 as the population of bacteria increases from 8 × 10^2^ (bacteria cm^−3^) to 8 × 10^6^ (bacteria cm^−3^). Similarly, we observe that the most probable Holder exponent *h*_0_(*q*) is shifting from 1.06 to 0.02 by increasing bacterial density from 8 × 10^2^ (bacteria cm^−3^) to 8 × 10^6^ (bacteria cm^−3^). The comparison shows that by increasing bacterial density, the width of the multi-fractal spectrum decreases; this implies that the bacterium motion dynamics transitions from a multi-fractal behaviour to a mono-fractal behaviour. Simply speaking, the bacterium oscillates more than exhibiting a directional motion. This transition translates into a reduction in *h*_0_(*q*). The lower *h*_0_(*q*) shows that the process is correlated, meaning that the underlying structure in bacterium motion becomes more regular by increasing the bacterial population. The increase in bacterial population makes their multi-fractal spectrum asymmetric, which captures the dominance of low- or high-fractal exponents with respect to medium fractal exponents. In the case of 8 × 10^6^ (bacteria cm^−3^) with chemoattractant in the environment, the right-skewed spectrum shows relatively strong weight for low-fractal exponents.

From figure 4*a*,*b*, we can conclude that adding chemoattractant to the environment has exactly the opposite effect on a single bacterium's motion compared to the effect of the increase in density. For example, we considered the two cases of bacterial population equal to 8 × 10^2^ (bacteria cm^−3^) without and with chemoattractant in the environment. The wideness (*W)* increases from 0.822 to 0.851 when we add chemoattractant to the environment. Similarly, the most probable Holder exponent *h*_0_(*q*) shifts from 0.78 to 1.06 by adding the chemoattractant. This implies that in an environment rich in chemoattractant, the multi-fractal spectrum related to a bacterium motion gets a wider shape with higher values of *h*_0_(*q*), and this is true for all the density cases we analysed except the highest density equal to 8 × 10^6^ (bacteria cm^−3^). In this case, because of the very high density of bacteria in the environment, the volume exclusion effects and the receptor saturations, there is no significant difference in *h*_0_(*q*) between the cases with and without chemoattractant and both of them are mono-fractal. In other words, adding chemoattractant to the environment impacts bacterial motion in a way that makes their motion more directional and less oscillatory. Figure 4*c*,*d* demonstrates the effect of obstacles in the environment for simulated bacteria (Note 8 in the electronic supplementary material discusses the importance of studying the effects of obstacles and also how they are considered in the computational model). We compared the case of bacterial density 8 × 10^4^ bacteria cm^−3^ without chemoattractant and with obstacles (*W* = 1.004 and *h*_0_(*q*) = 0.88) with the case of the same density with chemoattractant gradient and without obstacles (*W* = 0.6918 and *h*_0_(*q*) = 0.72). In an environment with obstacles, the multi-fractal spectrum shifts towards lower values of *h*_0_(*q*) and narrower spectrum widths. In such an environment, the bacterium has the tendency to oscillate more than to follow a directional motion.

### (b) Investigation of real *S. marcescens* swimming dynamics through *in vitro* experiments

We extract the *S. marcescens* motion trajectories from real experiments by the single particle tracking method and analyse them*.* The results from skewness analysis show the same nonlinear behaviour for the bacterium motion (electronic supplementary material, figure S1). This is in agreement with the previously presented results for simulated *S. marcescens,* meaning that the motion of *S. marcescens* is highly nonlinear in the environment.

Figure 4*e*,*f* shows the multi-fractal analysis for motion trajectories of *S. marcescens* from experiments *in vitro*. The results elucidate that *S. marcescens* has a multi-fractal motion in these environments. For the same population density, by adding chemoattractant to the environment the spectrum moves towards the right, which is a sign of multi-fractal behaviour. We observe a similar but not identical multi-fractal behaviour from real *S. marcescens* compared to simulated *S. marcescens*. The reason the results do not match exactly is that for the simulated *S. marcescens* we have control over most of the internal and environmental parameters. In contrast, real *S. marcescens* like other biological systems in nature show a higher degree of adaptation such as adjustment to environmental conditions, self-organization and complexity in their behaviour. Another reason for the difference between the simulation and experiment could be attributed to other forms of interactions that happen in the biological world and are not captured in the current computational model. However, if someone discovers new forms of interaction, they could be included in the computational model. This remains for future work.

## 4. Discussion

Bacteria like *S. marcescens* perform an efficient diffusive search (based on the status of their receptor signals) to find food in a complex environment [27]. In this study, we investigate the fractal characteristics of the bacterium motion while swimming in a three-dimensional environment. We first analyse the second- and third-order moment of the single bacterium motion trajectory with the ergodicity and nonlinearity tests under different conditions including bacterial density and chemoattractant distribution. These tests help building the bases for higher-order moment analysis of single bacterium motion through the MFDFA method to capture the entire spatial and temporal complexity of single bacterium motion. Based on MFDFA analysis, we show that bacterial swimming dynamics has multi-fractal characteristics. Moreover, we study how bacterial density and complexity of the environment (i.e. obstacles and chemoattractant) influence the multi-fractal characteristics of bacterial swimming dynamics. Consequently, our results contribute to the current state of knowledge by demonstrating that bacterial dynamics exhibits multi-fractal characteristics.

The effect of chemoattractant is one of the main environmental conditions affecting the multi-fractal characteristics of bacteria. Multi-fractal analysis shows that in the presence of an l-aspartate gradient in the environment, the multi-fractal spectrum gets wider and moves towards the right, meaning that bacteria tend to move directionally and oscillate less compared to the case without a chemoattractant gradient being present in the environment.

We also consider the effect of bacterial density as another important factor that affects the multi-fractal characteristics of bacteria. Our results show that increasing the density of bacteria makes the multi-fractal spectrum of their motion shift towards the left and transit to a mono-fractal behaviour. Moreover, further studies would be needed to relate these multi-fractal properties with the observed dependency between shear viscosity and density of the motile bacteria [89].

Previous research has shown that bacterial colonies display spatial fractal properties [90]. Ben-Jacob & Levine studied the spatial fractal properties of *Paenibacillus dedritiformis* bacteria colonies under the effect of the antibiotic Septrin [91]. They show that, in some patterns, the members of the colony stay closer together and form large vortices, which can increase the colony's ability to dilute the antibiotic with the lubricating fluid secreted by individual microbes; on the other hand, in some patterns their colony organizes into narrow and straight branches. This pattern happens when the food is scattered in the environment and will maximize bacterial contact with the limited nutrients in their environment. It is essential to emphasize on the point that, in contrast to previous research, we investigate the multi-fractal characteristics of the bacterium motion while swimming in a three-dimensional environment in this study.

This multi-fractal characteristic of the bacterium dynamics can get affected by continuous interactions among bacteria within the population. Moreover, this multi-fractal dynamic characterization of bacterial motion can allow us to quantify the effects of molecular (drugs) and gene-based therapies by investigating whether or not cells exhibit a directed movement as a result of the detection and transduction of external stimuli or continue to perform random wanderings. Interestingly, stem cell dynamics also exhibit a rich multi-fractal behaviour, although stem cells do not use flagella or cilia for locomotion. This suggests that this mathematical framework can be possibly used for identifying universal mathematical characteristics for cell motion irrespective of the size, scale and nature of interactions [92].

A comprehensive understanding of individual and collective movement of cells can be important for developing mathematical models of cell growth that can abstract and account for biologically relevant features such as cell density and volume exclusion effects, mechanical forces acting on cells surface, environmental conditions (e.g. pH, temperature), chemical factors (e.g. chemo-attractants, chemo-repellents), as well as factors that affect the internal state (e.g. cell-cell communication, mitotic phase, cell health state, cell age). For instance, the above-mentioned mathematical characterization can help decide the type of dynamical models (short-term memory versus long-term memory), the level of detail at which individual cells must be described (how much of the internal state needs to be modelled), or the effect of various external stimuli on an individual cell and collections of cells.

Overall, this newly identified mathematical property can enable the microscopic description of the motion of individual cells through concepts and tools from non-equilibrium statistical physics (e.g. multi-fractal master equation). Integrating over the population density and the characterized effect of internal and external stimuli could enable us to construct macroscopic models and propose control strategies for the population of cells in complex environments to reach specific targets [93–95]. An equally important research problem is to understand not only how the multi-fractal features allow for a compact multi-scale mathematical description, but also how they enable and simplify the controllability of complex systems [96,97]. This is left for future work.

## Supplementary Material

Supplemental material

## Data accessibility

Bacteria trajectory data (data are available in the following link: https://drive.google.com/file/d/0B449CRbDSlliM3JmUnBxSDg1OWs/view?usp=sharing).
