# Supplementary material for: Multi-fractal characterization of bacterial swimming dynamics: a case study on real and simulated Serratia marcescens
Source: Proc Math Phys Eng Sci. 2017 Jul 12;473(2203):20170154. doi: 10.1098/rspa.2017.0154 (PMC5549567; doi:10.1098/rspa.2017.0154)
Supplement: Supplemental material [file rspa20170154supp1.pdf]

Supplemental Material

Multi-Fractal Characterization of Bacterial Swimming Dynamics: A Case-study on Real and Simulated *Serratia marcescens*

Hana Koorehdavoudi[1,*], Paul Bogdan[2] , Guopeng Wei[3], Radu Marculescu[3], Jiang Zhuang[4]
Rika Wright Carlsen[4,5], and Metin Sitti[4,6]

[1]Department of Aerospace and Mechanical Engineering, University of Southern California, Los Angeles, CA 90089-1453, USA.
(koorehda@usc.edu)

[2]Department of Electrical Engineering, University of Southern California, Los Angeles, CA 90089-2560, USA.

[3]Department of Electrical and Computer Engineering, Carnegie Mellon University, Pittsburgh, PA 15213, USA.

[4]Department of Mechanical Engineering, Carnegie Mellon University, Pittsburgh, PA 15213, USA.

[5]Department of Engineering, Robert Morris University, Pittsburgh, PA 15108, USA

[6]Physical Intelligence Department, Max Planck Institute for Intelligent Systems, 70569 Stuttgart, Germany.

Note 1. Tests nonlinearity of motion based on Mardia's test

To check the nonlinearity of a bacterium motion we performed Mardia's test on the data set for bacteria trajectory. This method is based on multivariate extension of skewness measure. The bacterium motion is nonlinear if the skewness of trajectory of its motion is non-zero. This also demonstrates bacterium motion does not have a normal distribution [1, 2, 3].

Summary of Mardia's test: Based on Mardia's approach, the skewness of the data for a sample of $\{r_1, r_2, ... r_n\}$ of k dimensional vector relates to the third moment of the data set. The first step to find the third moment of data set is calculating the covariance matrix $\bar{\Sigma}$ from equation (1).

$$\bar{\Sigma} = \frac{1}{n}\sum_{j=1}^{n}(r_j - \bar{r})\,(r_j - \bar{r})^T \qquad (1)$$

In equation (1) and (2), $\bar{r}$ is the mean of the data set. In the next step we calculated skewness from equation (2)

$$skewness\ = \frac{1}{6n^2}\sum_{i=1}^{n}\sum_{j=1}^{n}[\,(r_i - \bar{r})^T\,\bar{\Sigma}^{-1}\,(r_j - \bar{r})]^3 \qquad (2)$$

Note 2. Henze-Zirkler's Multivariate Normality Test

Henze-Zirkler's Multivariate Normality Test is another approach to test the nonlinearity of data set. This test is based on a nonnegative functional distance that measures the distance between two distribution functions. We calculate this distance from equation (3)

$$D_b(P,Q) = \int_{R^2} |P(t) - Q(t)|^2 \times f_b(t)\, dt \quad (3)$$

P(*t*) is the characteristic function of a multivariable normal distribution and *Q*(*t*) is the empirical characteristic function of the data which we want to test, *Q*(*t*) should be centered and scaled to have the identity as the covariance matrix before comparison with normal distribution. $F_b(t)$ is the weight or kernel function calculated from equation (4).

$$f_b(t) = (2\times\pi\times b^2)^{\frac{-p}{2}} \times e^{\frac{-|t|^2}{2\times b^2}} \quad (4)$$

p is the number of variables and $|t| = (t't)^{0.5}$. The smoothing parameter b depends on the sample size n and can be calculated from equation (5).

$$b_p(n) = \frac{1}{\sqrt{2}}\left(\frac{2p+1}{4}\right)^{\frac{1}{p+4}} \times n^{\frac{1}{p+4}} \quad (5)$$

Henze-Zirkler statistic test know in literature as $T_{n,b}$ is as fallow:

$T_{n,b} = n \times (4 \quad 1\{S \text{ is singular}\} + W_{n,b} \quad 1\{S \text{ is nonsingular}\})$

where 1{.} stands for the indicator function and $W_{n,b}$ is weighted L^2 distance as follow:

$$W_{n,b} = \frac{1}{2}\sum_{j=1}^{n}\sum_{k=1}^{n} e^{-\frac{b^2}{2}\times D_{jk}} - 2(1+b^2)^{\frac{-p}{2}} \times \frac{1}{n}\sum_{j=1}^{n} e^{-\frac{b^2}{2\times(1+b^2)}\times D_j} + (1+2b^2)^{\frac{-p}{2}} \quad (6)$$

$D_{jk} = (X_j - X_k)\times inv(S)\times(X_j - X_k)'$

$D_j = (X - MX)\times inv(S)\times(X - MX)'$

X is data matrix, *MX* is sample mean vector and *S* is the covariance matrix normalized by *n*.

For a multivariate normal data, the test Henze-Zirkler statistic $T_{n,b}$ has a log-normal distribution. This test calculates the mean, variance and smoothness parameter. It will log-normalize the mean and variance and estimates the *p*-value [7, 8, 9].

This test has the following properties according to [8]

- Affine invariance
- Consistency against each fixed non-normal alternative distribution
- Asymptotic power against contiguous alternative of order $n^{-1/2}$
- Feasibility for any dimension and any sample size

Note 3. Royston's Multivariate Normality Test

To investigate the nonlinearity of a bacterium motion we performed Royston's marginal method which tests all the p variates for univariate normality with a Shapiro-Wilk statistic, then combines the p dependent tests into one omnibus test statistic for multivariate normality. Royston transforms the p-Shapiro-Wilk statistics into an approximate Chi-squared random variable, with e ($1<e<p$) degree of freedom. In this method, we estimate the degrees of freedom by taking into account possible correlation structures between the original p test statistics. If the data is multivariate normal, H function in equation (7) is approximately Chi-squared distributed.

$$H = e\,\frac{\sum_{j=1}^{p} R_j}{p} \tag{7}$$

Where

$$R_j = \{\varphi^{-1}\left[\frac{1}{2\varphi}\left\{-\frac{\left(1-W_j\right)^g - m}{s}\right\}\right]\}^2$$

$$e = \frac{p}{1+(p-1)\times mC}$$

W_j is the related value of the Shapiro-Wilk statistic for the jth variable in a p-variate distribution, g, m and s are calculated from polynomial approximation and φ^{-1} and $\varphi\,(.)$ are, respectively, the inverse and standard normal cdf. mC is an estimate of the average correlation among the R_j's. This Chi-Square distribution is used to obtain the critical or p-value for the MNV test [10-17].

Note 4. Doornik-Hansen Omnibus Multivariate Normality Test

Doornik-Hansen Omnibus Multivariate Normality Test is the multivariate version of the univariate omnibus test to check normality of bacterium trajectory based on the transformed skewness and kurtosis [4, 5, 6]. We used the correlation matrix and the diagonal matrix of reciprocals of the *p* standard deviations and transformed a multivariate normal into independent standard normal by equation (8).

$$st = Z \times V \times L^{-\frac{1}{2}} \times V' \tag{8}$$

In this equation *Z* is the standard normalized data matrix, *V* is the eigenvector matrix and *L* is the eigenvalues diagonal matrix. In this method skewness is transformed using the D'Agostino procedure and kurtosis is transformed from a gamma distribution to a chi-square. We calculated Doornik-Hansen statistic from equation (9), which approximates to a chi-square with $2p$ degrees of freedom.

$$DH = z_1 \times z_1' + z_2 \times z_2' \tag{9}$$

Table 1. Summary of linearity tests on a single bacterium trajectory from simulated bacteria.

**Method**	**Variables**	**Result**
Henze-Zirkler's Multivariate Normality Test	Henze-Zirkler lognormal mean: -0.1520616<br>Henze-Zirkler lognormal variance: 0.1428457<br>Henze-Zirkler statistic: 45.5201260<br>*P*-value associated to the Henze-Zirkler statistic: 0.0000000<br>With a given significance = 0.050	Data analyzed do not have a normal distribution (*P*<*alpha*)
Royston's Multivariate Normality Test	Royston's statistic: 241.735218<br>Equivalent degrees of freedom: 1.903974<br>*P*-value associated to the Royston's statistic: 0.000000<br>With a given significance = 0.050	Data analyzed do not have a normal distribution (*P*<*alpha*).
Doornik-Hansen Omnibus Multivariate (Univariate) Normality Test	Asymptotic statistic: 284.3903<br>*P*-value associated to the asymptotic statistic: 0.0000<br>With a given significance = 0.050<br>Omnibus Doornik-Hansen statistic: 981.4541<br>*P*-value associated to the Omnibus Doornik-Hansen statistic: 0.0000<br>With a given significance = 0.050	Data analyzed do not have a normal distribution (*P*<*alpha*).

These result are based on different tests on the bacterium trajectory from simulated bacteria with density of 8×10^2 (Bacteria/cm3) in a cubic environment with size 5mm×5mm×5mm without chemoattractant in the environment. Number of variables in all the tests are 3 as the bacterium trajectory is in 3D environment, and the sample size is 1001. All the tests results are in agreement that the bacteria motion does not have normal distribution; hence, bacterium has nonlinear motion.

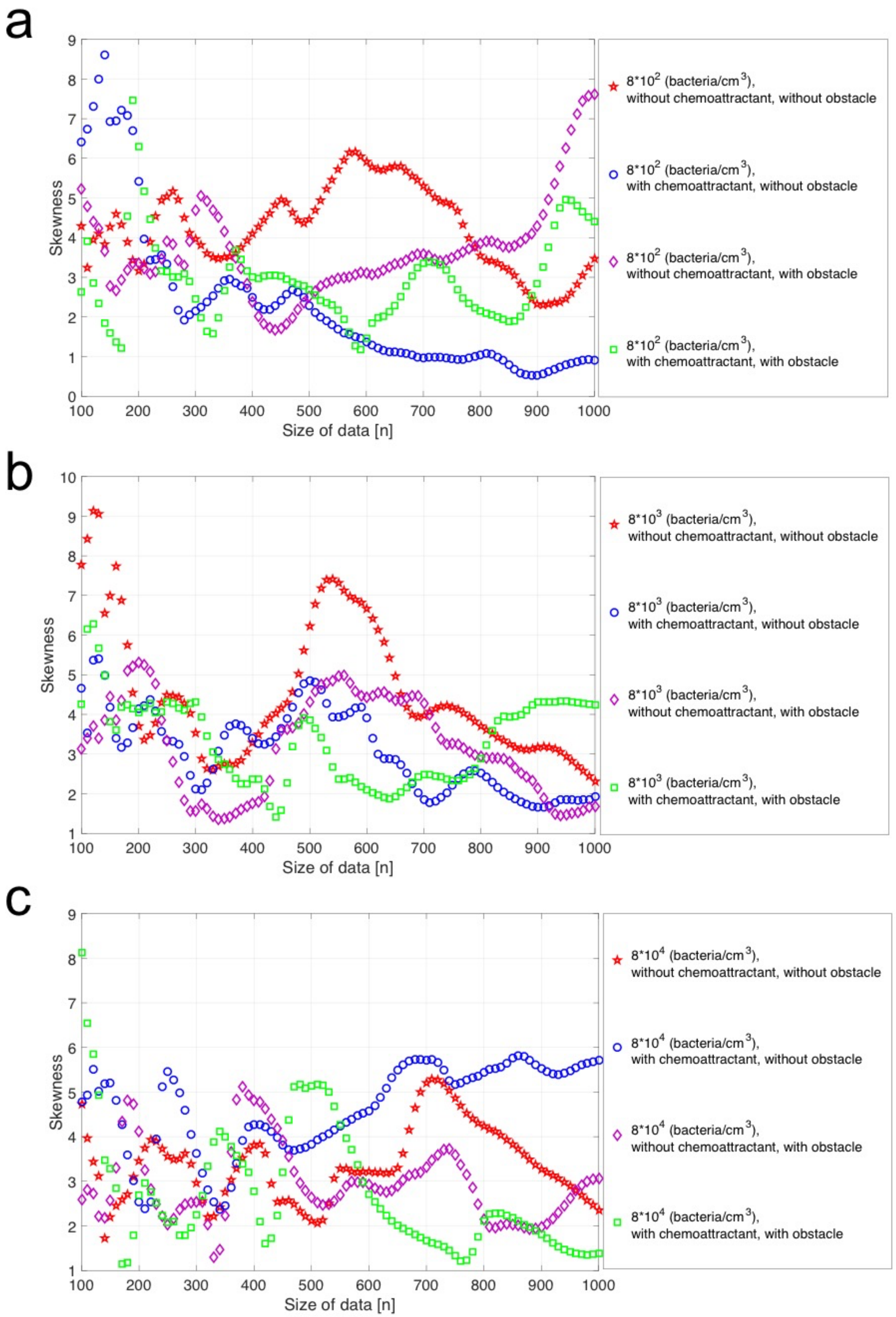

Figure S1. Skewness plots for some of the simulation bacteria trajectories. a) 8×10^2 (bacteria/cm^3) in the cubic environment, with/out linear gradient of chemoattractant in *y* direction, with/out obstacles on the bacteria way. **b)** 8×10^3 (bacteria/cm^3) in the cubic environment, with/out linear gradient of chemoattractant in *y* direction, with/out obstacles on the bacteria way. **c)** 8×10^4 (bacteria/cm^3) in the cubic environment, with/out linear gradient of chemoattractant in *y* direction, with/out obstacles on the bacteria way. In all the cases skewness is unequal to zero meaning bacterium displacement does not have normal distribution; therefore, bacterium motion is nonlinear.

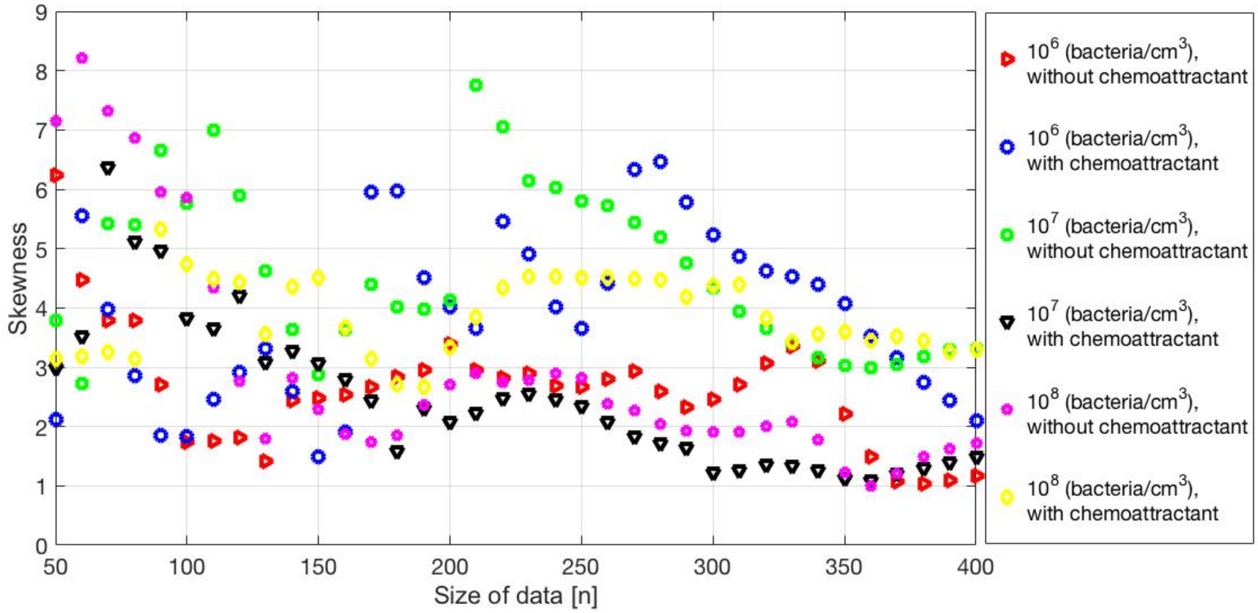

Figure S2. Skewness plots for experimental *S. marcescens* trajectories. Skewness plots for different bacteria density cases in vivo environment, with/out linear gradient of chemoattractant in *y* direction. In all the cases skewness is unequal to zero meaning bacterium displacement does not have normal distribution; therefore, bacterium motion is nonlinear.

Note 5. Multi-fractal detrended fluctuation analysis (MFDFA).

We performed MFDFA to investigate the higher order moments of a bacterium trajectory of motion to shed light on the underlying structure in bacteria motion [54]. In general, the dynamics of a time series from a stochastic process is fractal if it displays self-similarity of specific features on different scales. The fractal dimension represents a measure for these unique features (e.g., irregularities or patterns) in the stochastic process. Two major types of fractals are known as mono-fractal and multi-fractal. mono-fractal dynamics can be characterized by a single fractal dimension. In other words, if the probability distribution function of the stochastic process at two different scale is self-similar and related to each other via a power law relationship, the stochastic process is mono-fractal. In contrast, a multi-fractal stochastic process is characterized by a series of fractal dimensions. The MFDFA analysis helps to verify whether the bacterium motion is multi-fractal or mono-fractal by computing the generalized Hurst exponent as a function of the higher order moment and the multi-fractal spectrum. We explain the MFDFA analysis of the time series in the following steps.

The first step in MFDFA is checking that the time series has a random walk like structure. If the time series (x) has a noise like structure, it should be converted to a random walk type time series using equation (10).

$$Y(i) = \sum_{k=1}^{i}[x_k - \langle x \rangle] , \qquad i = 1, \ldots, N \tag{10}$$

In equation (10), $\langle x \rangle$ is the mean of time series.

Next, based on scaling size s, we divided the time series Y into N_s non-overlapping subintervals using equation (11). This equation returns the integer part of the division.

$$N_s = int\left(\frac{N}{s}\right) \tag{11}$$

Since the length of the time series N is not always an exact multiple of the scaling size s, a short part of the time series at its end will remain after dividing the time series Y into N_s non-overlapping subintervals. To consider the remaining part of the time series from its end and avoid disregarding it, we repeated the same division procedure starting from the opposite end of the time series. In other words, we are dividing the time series into N_s non-overlapping subintervals from the end point of the time series, and this time a short part of the time series in the beginning of the time series will remain. Therefore, we obtain $2N_s$ segments overall [55].

The next step is computing the local Root-Mean-Square variation of the time series in each segment using equation (12) and (13). This quantifies the local fluctuations in the time series.

$$F^2(s, \nu) = \frac{1}{s}\sum_{i=1}^{s}\{Y[(\nu-1)s + i] - y_\nu(i)\}^2 \qquad \nu = 1, \ldots, N_s \tag{12}$$

$$F^2(s, \nu) = \frac{1}{s}\sum_{i=1}^{s}\{Y[N - (\nu - N_s)s + i] - y_\nu(i)\}^2 \qquad \nu = N_s + 1, \ldots, 2N_s \tag{13}$$

In equation (12) and (13), $y_\nu(i)$ is the polynomial (e.g. linear, quadratic, cubic or higher order polynomial) fitted to each segment ν. In our analysis we considered a linear fit.

In the next step we averaged over all segments to quantify the qth order fluctuation function. In other words, we calculated the mean of q-order RMS for corresponding scaling size s and order q from equation (14).

$$F_q(s) = \{\frac{1}{2N_s}\sum_{\nu=1}^{2N_s}[F^2(s,\nu)]^{\frac{q}{2}}\}^{\frac{1}{q}} \quad (14)$$

We repeated all the steps for several values of the time scales s. Then we determine the scaling behavior of the fluctuation functions by analyzing log-log plots $F_q(s)$ versus s for each value of q. If the time series has a long-range power-law correlation, equation (15) will be valid. In other words, mean of q-order RMS ($F_q(s)$) increases as a power-law for large values of s.

$$F_q(s) \approx s^{H(q)} \quad (15)$$

For simplicity we considered that the length N of the time series is an integer multiple of the scale s, meaning $N_s = N/s$:

$$\sum_{\nu=1}^{2N_s}[F^2(s,\nu)]^{\frac{q}{2}} \sim s^{q\times H(q)-1} \quad (16)$$

We call the function $H(q)$ generalized Hurst exponent which captures the fluctuations in the data set. Generally, exponent $H(q)$ may depend on q. For Mono-fractal time series, $H(q)$ is independent of q, since the scaling behavior of the variance $F^2(s,\nu)$ is identical for all the segments ν. If small or large fluctuations scale differently, there will be significant dependence of $H(q)$ on q, which means the time series is multi-fractal. Therefore, we can use generalized Hurst exponent to investigate whether the time series is mono-fractal or multi-fractal. Moreover, the generalized Hurst exponent allows us to compute the Hurst parameter and discriminate between short-range and long-range memory effects. For instance, if the Hurst exponent equals 0.5 then we can state that the dynamic is governed by short-range memory or Markovian models. In contrast, if the Hurst exponent ranges between 0.5 and 1, then the dynamics has long-range memory characteristics and non-Markovian dynamical models are needed.

Another way to distinguish between mono-fractal vs. multi-fractal time series is to first convert $H(q)$ to the scaling exponent $\tau(q)$ (defined by equation (17)). And then convert $\tau(q)$ to Holder exponent (i.e. singularity strength) $h(q)$ and singularity spectrum D_q (defined by equation (18) and (19)) via a Legendre transform [55].

$$\tau(q) = q\times H(q) - 1 \quad (17)$$

$$h(q) = \tau'(q) \quad (18)$$

$$D_q = q\times h(q) - \tau(q) \quad (19)$$

In the case of mono-fractal time series, the scaling exponent $\tau(q)$ has a linear dependency with q. The linear q-dependency of $\tau(q)$ leads to a constant $h(q)$ of these time series because $h(q)$ is the tangent slope of $\tau(q)$. The constant $h(q)$ reduces the multi-fractal spectrum to a small arc for the mono-fractal time series. In contrast, the multifractal time series has scaling exponents $\tau(q)$ with a curved q-dependency and, consequently, a decreasing

singularity exponent $h(q)$. The resulting multi-fractal spectrum is a large arc where the difference between the maximum and minimum $h(q)$ are called the multi-fractal spectrum width. Thus, the width and shape of the multi-fractal spectrum is able to classify a wide range of different scale invariant structures of time series [54].

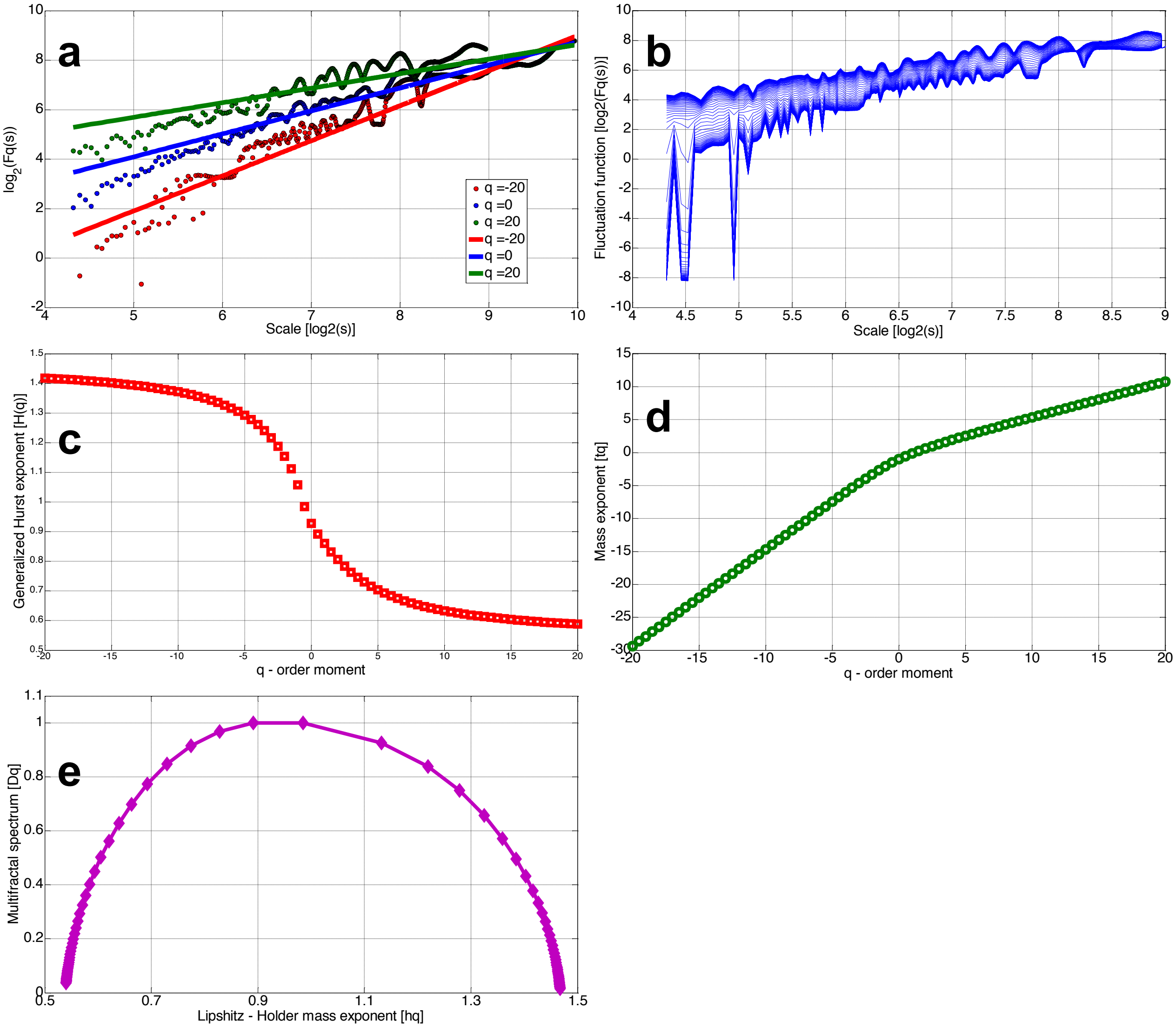

Figure S3. Multifractal detrended fluctuation analysis results. Results related to MFDFA analysis for simulated bacteria for the density of 8×10^2 (bacteria/cm^3) in a cube of 5mm × 5mm × 5mm without any chemoattractant and obstacles in the environment. **a)** Scaling function Fq as a function of scale s for q = -20, 0, 20. **b)** Generalized Hurst exponent as a function of the *q*–th order moment. **c)** Fluctuation function *Fq*(*s*) as a function of scale *s* and for various *q*-th order moment. **d)** Mass exponent function as a function of *q*–th order moment. **e)** multifractal spectrum as a function of the Lipschits Holder mass exponent. All the results from MFDFA analysis shows the bacterium motion is multifractal in this case.

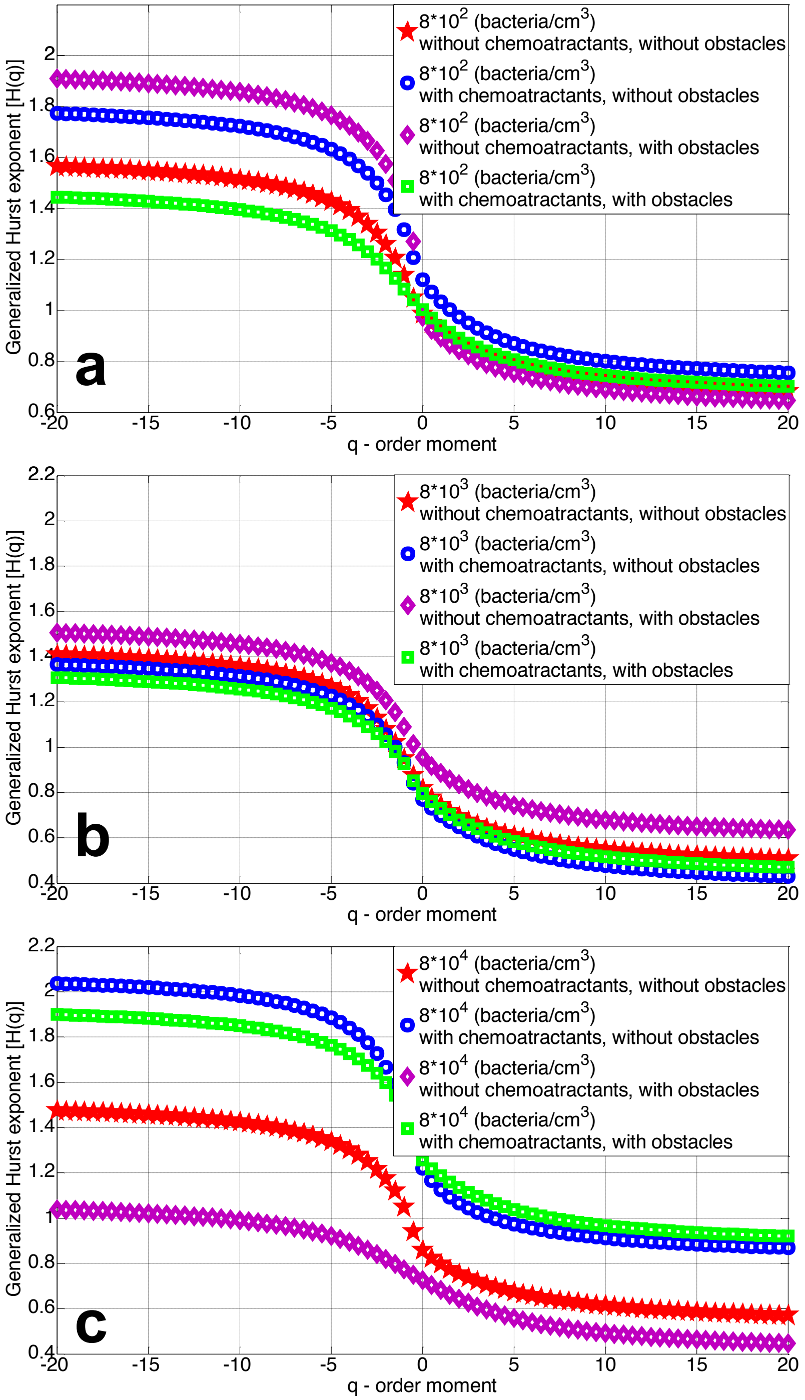

Figure S4. Generalized Hurst exponent plots for the simulated bacteria trajectories. a) 8×10^2 (bacteria/cm^3) in the cubic environment, with/out linear gradient of chemoattractant in y direction, with/out obstacles on the bacteria way. **b)** $8{\times}10^3$ (bacteria/cm^3) in the cubic environment, with/out linear gradient of chemoattractant in y direction, with/out obstacles on the bacteria way. **c)** $8{\times}10^4$ (bacteria/cm^3) in the cubic environment, with/out linear gradient of chemoattractant in y direction, with/out obstacles on the bacteria way. The results show increasing the bacteria density make them less directional motile and they tend to oscillate more. Adding chemoattractant to the environment makes the bacteria more directional motile towards the increasing gradient of chemoattractant and oscillating less. Obstacles in the environment make bacteria to oscillate more to prevent hitting them.

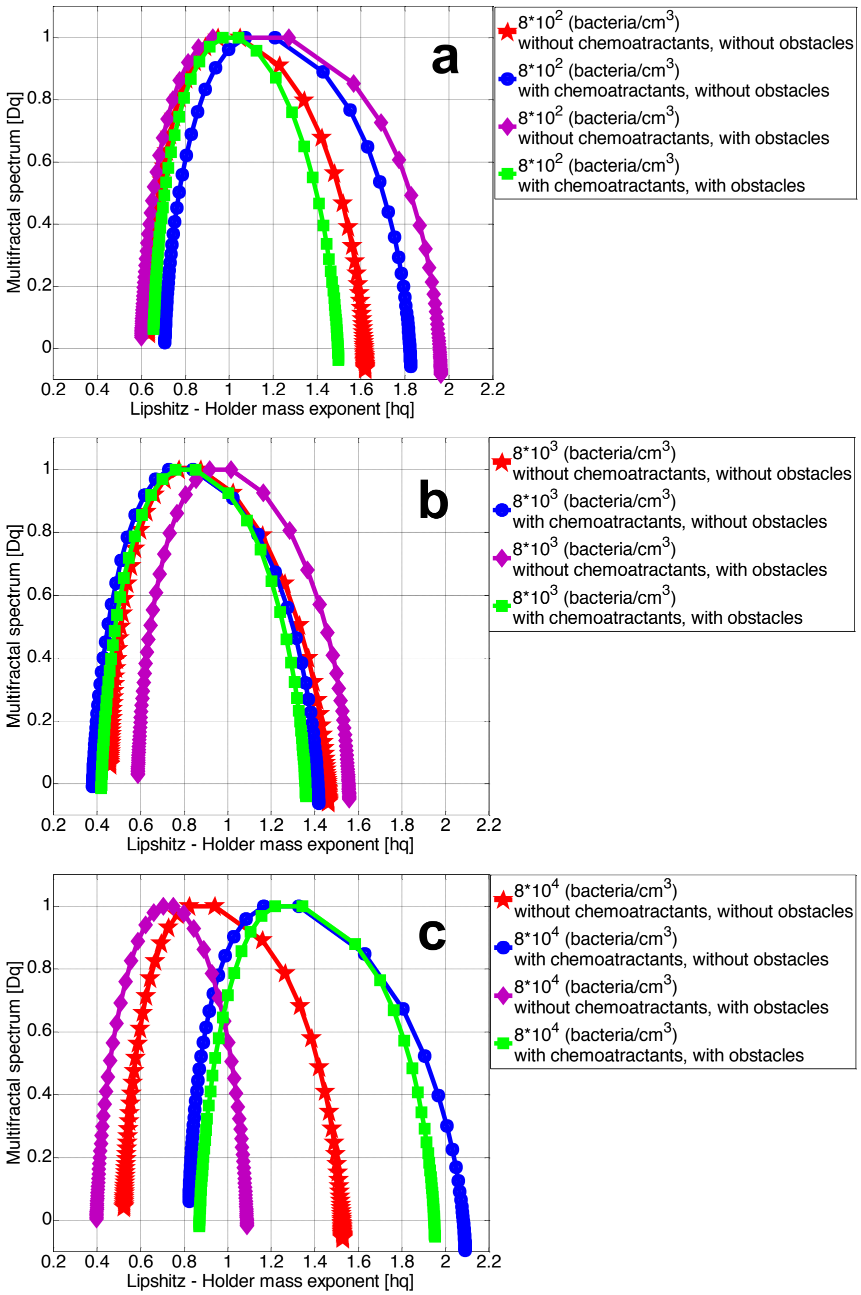

Figure S5. Multifractal spectrum plots for the simulated bacteria trajectories. a) $8x10^2$ bacteria/cm^3 in the cubic environment, with/out linear gradient of chemoattractant in *y* direction, with/out obstacles on the bacteria way. **b)** $8x10^3$ bacteria/cm^3 in the cubic environment, with/out linear gradient of chemoattractant in *y* direction, with/out obstacles on the bacteria way. **c)** $8x10^4$ bacteria/cm^3 in the cubic environment, with/out linear gradient of chemoattractant in *y* direction, with/out obstacles on the bacteria way. The results show increasing the bacteria density make them less directional motile and they tend to oscillate more. Adding chemoattractant to the environment makes the bacteria more directional motile towards the increasing gradient of chemoattractant and oscillating less. Obstacles in the environment make bacteria to oscillate more to prevent hitting them.

Note 6. Mathematical Modeling Used in BNSim

To characterize the dynamics of real bacteria *S. marcescens* we developed a simulation environment (namely BNSim). BNSim is an open source (source code can be downloaded from http://www.ece.cmu.edu/~sld/bnsim/index.html), parallel multi-scale stochastic modeling platform integrating three stochastic simulation methods (i.e., Gillespie's exact stochastic simulation algorithm [19], stochastic differential equations [19], and a new hybrid simulation algorithm based on the hierarchy and dynamics of biochemical systems) with genetic circuits and chemotaxis pathway models in a complex 3D environment. Specifically, to simulate the chemoreceptors, we use the Monod-Wyman-Changeux model in which the receptor homo-dimers assemble into fully cooperative signaling teams that switch rapidly between active and inactive states. The methylation kinetics is based on the well-known Barkai and Leibler model [20] for a near perfect adaptation system. For the signal transduction from chemoreceptor to the flagella motor regulator Yp, the concentration of phosphorylated CheYp is assumed to be proportional to the kinase activity without considering the nonlinear dependence [21]. Finally, we use a two-state model to describe the motor behavior of bacteria, which sets the clockwise (CW) and counterclockwise (CCW) states in two potential wells. The transition rates have been fitted to experimental data obtained from [22].

To better clarify the validity of our model, in what follows, we also provide a more detailed description of the full chemical pathway model of bacteria used in our BNSim simulation environment. In general, the chemotaxis pathway of *S. marcescens* has three components: the cooperative chemoreceptor, the phosphorylation pathway, and the flagellar motor [24, 25-27, 37]. In what follows, we describe the three components of the chemotaxis pathway.

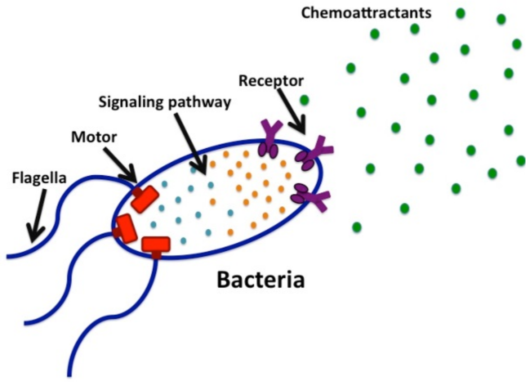

Figure S6. bacteria chemical pathway in an environment with chemoattractant

a. Cooperative Chemoreceptor Cluster

To describe the cooperativity of chemoreceptors, we use the latest MWC model that captures the essential features of bacteria chemotactic activity and adaptation [37]. More specifically, each functional methyl-accepting chemotaxis protein (MCP) receptor complex can be either in the active or the inactive states, which

are determined by a free energy difference F(m, $[L]$), where m is the methylation level of the receptors, $[L]$ is the concentration of the ligand, and N is the number of receptor dimers in a MCP complex. We use $N = 6$ for Tar receptors in a complex; the average activity of the receptor can be expressed as [23, 28]:

$$a = (1 + \exp(F(m, [L])))^{-1} \tag{20}$$

According to the MWC model, the free energy difference can be written as:

$$F(m, [L]) = f_m(m) - \ln\left(1 + \frac{[L]}{K_a}\right) + ln\left(1 + \frac{[L]}{K_i}\right) \tag{21}$$

In equation (21) $f_m(m)$ is the methylation level dependent free energy difference, and K_a and K_i are the dissociation constants of the ligand to the active and the inactive receptors, respectively. For the binding of L-aspartate onto the Tar receptors, we use the values fitted to *in vivo* FRET (fluorescence resonance energy transfer) data [23], namely, $K_{\{a\}} = 3$ mM and $K_{\{i\}} = 18.2\ \mu M$. The impact of receptor methylation on its free energy is considered to be a linear function of m as suggested by recent experimental work [29, 30]:

$$f_m(m) = \alpha(m_0 - m) \tag{22}$$

In equation (22) $\alpha = 1.7$ and $m_0 = 1$.

The methylation kinetics is based on the Barkai and Leibler model for a near perfect adaptation system [39]. More precisely, the methylation kinetics is assumed to have a linear form:

$$\frac{dm}{dt} = k_R(1 - a) - k_B a \tag{23}$$

In equation (23) $k_R = k_B$ are the methylation and demethylation rates, respectively.

b. Phosphorylation Relay and Flagellar Motor

An active receptor enhances the autophosphorylation of the receptor-associated kinase CheA, which transmits the signal to the flagellar motors by the phosphorylation of a diffusive response regulator CheY. In most chemotaxis full pathway models, the concentration of phosphorylated CheYp is assumed to be proportional to the kinase activity, $[Y_P] = \beta a\ (t)$, without considering the nonlinear dependence [28].

Thermal fluctuations and upstream signaling cause the flagellar motor to spontaneously change between CCW and CW states. To model the state transition, the SPEC model uses a Hill function to calculate the probability of tumble with $[Y_P]$, and assumes an average fixed tumble time $\tau = 0.2$ s [23]. Specifically, when a bacterium is running, the probability of the cell going into a tumble state is $p(a) = \tau_1^{-1}(\frac{a}{a_{\frac{1}{2}}})^H$, where $a_{1/2}$ is a fitted constant, and H is the Hill coefficient of the motor response function. However, under some gradient conditions, the bacteria flagellar motor may stay in the tumble state for a time significantly shorter or longer than the average

value, which biases the swimming dynamics considerably [40]. Therefore, we adopt a two state potential well model to describe the motor behavior of bacteria, which sets their two states in two potential wells.

The energy barriers of CCW to CW and CW to CCW transitions are $G_0([Y_P])$ and -$G_0([Y_P])$, with transition rates k^- and k^+, respectively [22]:

$$G_0([Y_P]) = \frac{g_0}{4} - \frac{g_1}{2}\left(\frac{[Y_P]}{K_D+[Y_P]}\right) \tag{24}$$

$$k^+ = \omega_0 \exp\left(G_0([Y_P])\right) \tag{25}$$

$$k^- = \omega_0 \exp\left(-G_0([Y_P])\right) \tag{26}$$

Where parameters $\omega_0 = 1.3\ s^{-1}$, $g_0 = g_1 = 40\ k_B T$, and $K_D = 3.06\ \mu M$are chosen to fit the experimental data.

Bacteria Transport Model:

In a bounded channel with chemical gradient, change in the motion of single bacteria leads to a biased distribution of bacterial density. Based on probabilistic modeling of single bacteria, the model described in this section relates the population kinetics to the motility parameters of individual cells. The chemotactic velocity (V_C) introduced in the model quantifies the effect of chemotaxis on the bacterial population transport; by construction, V_C is directly related to the tumble rate bias of individual bacteria. Therefore, by extracting V_C from this model and determining it through tracking of single bacteria, this paper quantifies chemotaxis from the measurement of individual cells, as opposed to most recent studies, which have made measurements at the population level [23, 31, 32, 38].

In an environment with a one-dimensional (along *x*-direction) chemoattractant gradient, the transport kinetics of bacteria density can be described with the following equation [33]:

$$J_x = -\mu \frac{\partial B(x,t)}{\partial x} + V_C B(x,t) \tag{27}$$

In equation (27) J_x is the density flux of bacteria through a slice that is perpendicular to the *x* axis, μ is the motility coefficient, $B(x,t)$ is the bacteria density, and V_C is the chemotactic velocity as previously discussed. The motility coefficient, μ, similar to the diffusion coefficient, is a proportionality measure between the bacterial density flux and the gradient of the bacterial density. Bacteria transport in the channel is subject to two effects: the diffusivity motility and the chemotactic drift, which are described by the first and second terms on the right hand side of Equation 27, respectively. The diffusivity motility keeps the transport bacteria down the gradient of bacterial population density, while the chemotactic velocity pumps bacteria up the chemoattractant gradient. From the probabilistic modeling of individual bacteria, the motility coefficient and chemotactic velocity can be expressed as follows [34, 37]:

$$V_C = \frac{2(p^- - p^+)}{3(p^- + p^+)} v \tag{28}$$

$$\mu = \frac{2v^2}{3(p^- + p^+)(1 - cos < \phi >)} \tag{29}$$

In equation (28) and (29), p^+ and p^- are the average tumble rates when bacteria travel up and down the chemical gradient, respectively, v is the average 3D swimming speed of bacteria, and $< \phi >$ is the average tumble angle, which is defined as the swimming direction change during a tumble. Instead of a simple model based on average rate processes, models based on random walk theories that consider distributions of running and tumbling durations are also available [35, 36]. These advanced models could provide a more accurate characterization of single bacteria motility and population transport if both the run and tumble durations of bacteria can be measured experimentally in the future.

We have performed the following tests to confirm that simulated bacteria in BNSim exhibit similar characteristics of real bacteria:

1. In BNSim we have calibrated the model of a bacterium using experimental data reported for real bacteria [21]. Figure S7 shows the calibration results and the validity of our model. For instance, the experimental results for normalized initial receptor activity [51] are shown in the upper Fig. S7.b, while the BNSim simulation results for the wild type bacteria is shown in the figure S7.b below. Exact receptor activity curve ensures that bacteria respond to external stimuli correctly. Figure S7.c presents CW bias and switching frequency of a flagella motor as a function of CheY-P concentration. The experimental results are shown on the left [40], while the BNSim simulation results are shown on right. Moreover, figure S7.d exhibit run intervals of bacteria simulated in BNSim obey a power-law distribution in nutrient-free environments [52].

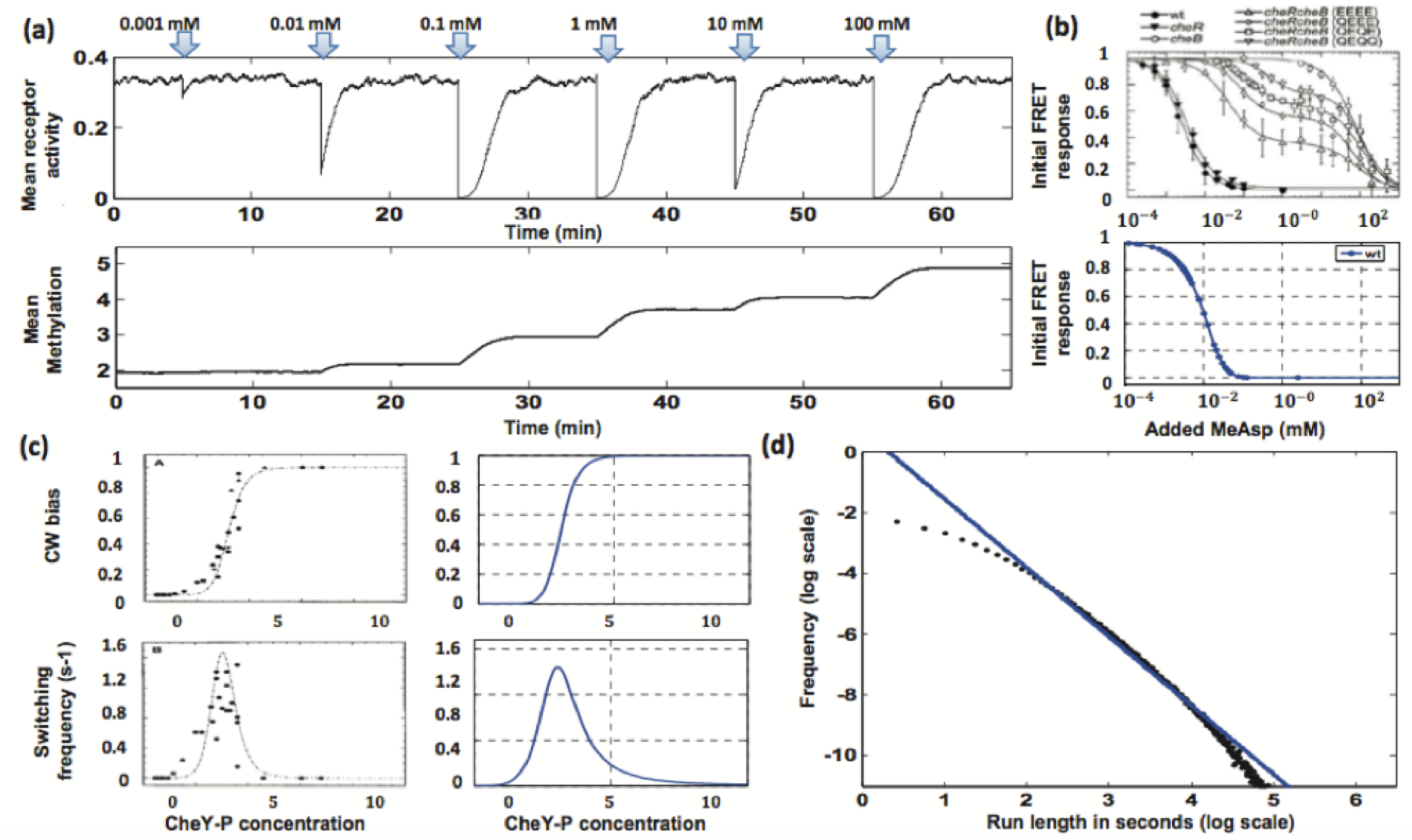

Figure S7. Single cell model calibration. (a) Bacteria precise adaptation. The bacterial chemotaxis network is remarkably sensitive to small changes in chemical concentrations over a wide range of ambient concentrations. The figure is obtained by averaging BNSim simulation results over 370 receptor clusters. (b) Normalized initial receptor activity. The experimental results [51] are shown in the upper figure, while the BNSim simulation results for the wild type bacteria are shown in the figure below. Exact receptor activity curve ensures that bacteria respond to external stimuli correctly. (c) CW bias and switching frequency of a flagella motor as a function of CheY-P concentration. The experimental results are shown on the left [40], while the BNSim simulation results are shown on right. (d) BNSim simulation results show bacteria run intervals obey a power-law distribution in nutrient-free environments [52].

2. Figure S8 shows the simulation results obtain with BNSim for synchronization process on bacteria swarm ring that expand radially. The simulation result matches experimental observation reported in Brenner and Berg [21, 41].

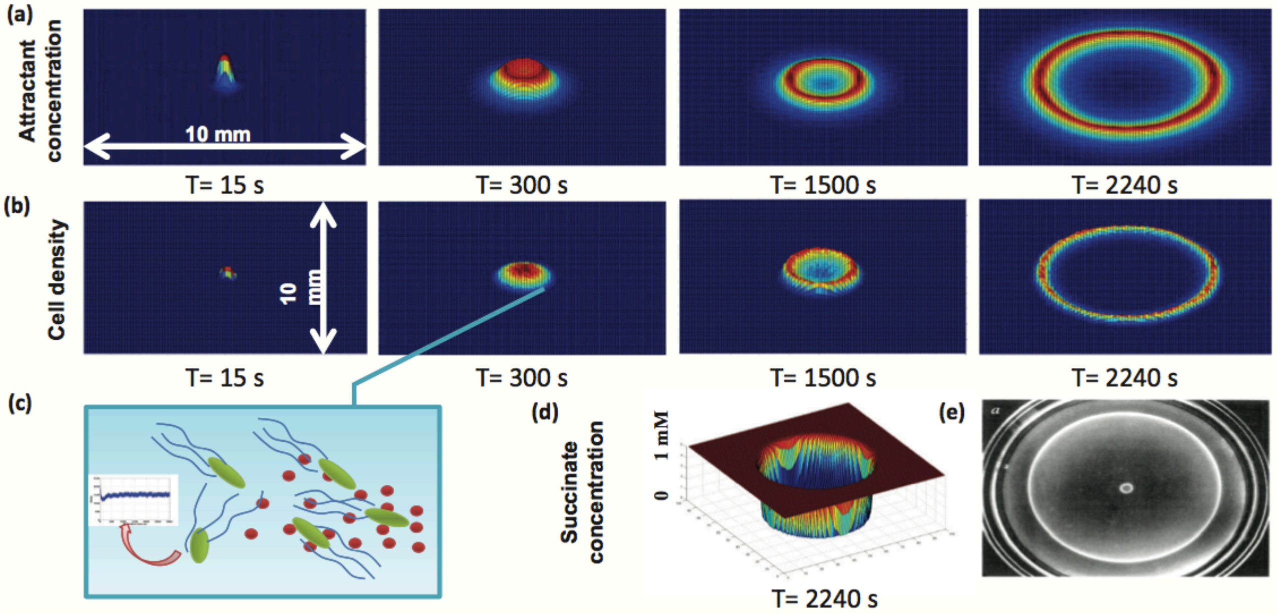

Figure S8. Swarm ring formed by chemotactic bacteria strains (simulation results obtained from BNSim using the model in [46]) (a) Attractant concentration as a function of time. Attractant molecules are excreted by bacteria without external gradient. Bacteria convert substrate succinate to attractant by the enzyme aspartate from fumarate and ammonia. However, at low succinate concentration, bacteria uptake aspartate molecules which creates an attractant gradient. BNSim is able to unveil the subtle actions of real chemicals involved in wet experiments. (b) Cell density of bacteria population as a function of time. The distribution of chemotactic bacteria population follows the gradient of the diffusing attractant ring. Since cell division has a much larger time scale, it only weakly affects the formation of swarm ring. (c) A zoomed-in view of a group of bacteria at the edge of the swarm ring. BNSim allows researchers to check the fine-grain details anytime, such as the molecular dynamics of any individual bacterium. This is a unique advantage of our agent-based approach. (d) Succinate concentration at time T = 2240 s. As shown, the substrate succinate inside the swarm ring is nearly depleted. Therefore, the aspartate cannot be produced inside the ring. (e) Experimental observation of swarm ring at low succinate concentration.

Note 7. Volume exclusion effects considered in our model

The dependency of a single bacterium behavior on density of the whole swarm is mainly due to volume exclusion effects, their collective motion and chemical interaction between bacteria known as autochemotaxis. Based on volume exclusion effect, in nature, no two bacteria can occupy the same space at the same time in the environment. To capture this behavior in our simulations we assign 3 dimensional locations for the center of the volume of each bacterium. In each step of the simulation, we determine the next location of each bacterium in the swarm based on its chemical pathway and autochemotaxis between them; meanwhile, we check if there are any two bacteria, which have the same location in the environment at the same time step. When two bacteria run into each other, they both have a certain probability following a Gaussian distribution to tumble and choose a new forward direction. This approach is based on the experimental observation. If there is no such case that two bacteria run into each other, we update the location of all the bacteria in the swarm and continue the simulation in the next time step.

The differences between volume exclusion model and point-like model in BNSim have been shown in previous research [53]. In essence, the volume exclusion is a major source of bacteria non-linear behavior at high cell-density. In our simulations we consider each bacterium occupy a specific space based on its volume, and we check for non-overlapping cells based on the space not only center of mass.

Note 8. Obstacles considered in our model

In realistic scenarios of using bacteria, the swarm dynamics will be affected by collisions with existing obstacles. Consequently, we consider the effect of obstacles in the mathematical modeling of the swarm of bacteria. In our considered simulation setup, we divide the environment into 10^6 smaller cubes, each of size $50\times50\times50$ μm^3. We consider 0.01 percent of the total number of cubes obtained after tessellation of the environment to represent obstacles, and choose the location of obstacles to follow a uniform distribution within the environment (Fig. 1a). The way we implement the obstacles in our simulation is by making these small cubes to be impenetrable, thus when a bacterium approach one of these cubes it cannot continue swimming and so it chooses a new direction for runs.

Another reason to use small cubes is to speed up the calculation in simulations. Precisely, one data element associated with each small cube stores all references of living and non-living objects in the local environment of that cube; these data elements can therefore be accessed very efficiently according to their indexes as a function of their absolute position in the global environment. This way, we can ensure that interactions between bacterium and environment, also interactions among bacteria themselves can be efficiently simulated. Of note, to ensure the correctness of the result, access to each cube needs to happen in a mutually exclusive manner so each bacterium needs to obtain the corresponding mutex beforehand. Therefore, the small cubes help to reduce the computational complexity, so we only need to check particle pairs for collision condition inside the same cube and neighboring cubes.

Note 9. Similarities between *S. marcescens* and *E. coli*

Similarities between *S. marcescens* and *E. coli* have been observed both in their morphologies and their responses to environmental stimuli. Both strains rely on the rotation of their peritrichous flagella to swim around. When in a free swimming state, their trajectories feature periods of directional runs (~ 1 s) interrupted by much smaller periods of tumbles (~ 0.1 s). The run and tumble states correspond to the bundled and unbundled states of their flagella, respectively. The tumble rate is a critical parameter for characterizing their motion, and both bacteria have been observed to maintain a similar approximated average tumble rate, between 1.2 s^{-1} and 1.4 s^{-1} from the observation[42, 43]; the average swimming speeds for both bacterial strains range from 20 to 50 μm/s [42, 44].

In addition to the resemblance in morphology and motility, the two species show nearly the same response to canonical chemoattractant, such as L-aspartate, as shown in [42] and [45]; and their chemotactic signaling pathways can be modeled in the same way to predict their behavior [45]. It has been shown that Tsr and Tar chemical receptors on the cell membrane are responsible for sensing chemical gradients, as well as pH and temperature gradients, and initiating signaling pathways that lead to a change in the rotation of the flagellar motors. This, in turn, drives the chemotactic behavior and responses to pH and temperature gradients. Given the nearly identical response to temperature and chemical gradients between *S. marcescens* and *E. coli,* it has been hypothesized that they share similar chemical sensors and signaling pathways [42].

Note 10. Autochemotaxis considered in our model

We investigate bacteria dynamics motion under realistic conditions such as chemical interactions among bacteria. Autochemotaxis is an example of chemical interaction between bacteria. Autochemotaxis happens when bacteria convert substrate succinate molecules in the environment into chemoattractant aspartate molecules and excrete them in the environment. Once the succinate gets depleted, bacteria start consuming the chemoattractant aspartate excreted by themselves; this way, they may get some information about the population of bacteria around them [41, 47, 48, 49].

In our BNSim simulations, we consider that the diffusion coefficient of succinate is the same as aspartate which is equal to 890. The initial concentration of Succinate is 5 μm, the production rate of Aspartate is $2e^{-3}(1/s)$. The concentration of Aspartate follows equation (30).

$$\frac{d[asp]}{dt} = k_{asp} \times \frac{[succinate]}{([succinate]+K_s)} \quad , \qquad K_s = 1\ \mu m \tag{30}$$

The consumption of Aspartate is modeled as presented in equation (31).

$$\frac{d[asp]}{dt} = -U_{asp}\ , \quad U_{asp} = 1.5e^{-6}(1/s) \tag{31}$$

we assume that bacteria start to consume attractant aspartate 2 minutes after being in a low succinate environment [46]. Figure S8 shows the simulated synchronization process of a bacteria swarm ring that expands radially that we performed to validate autochemotaxis model in BNSim [21]. We reproduced this experimental phenomenon with BNSim by using the theoretical model proposed by Brenner et al (see also the experimental results in Fig. S8(e) by Brenner and Berg [41]). When bacteria are initially located in the center of a 3D space, they start excreting chemoattractant aspartate by converting the substrate succinate molecules in the environment into chemoattractant molecules. Once the succinate gets depleted, bacteria start consuming the chemoattractant aspartate excreted by themselves (T = 300s in Fig. S8(a)). Therefore, the aspartate concentration in the inner region of the ring becomes lower than the concentration outside the ring. This way, bacteria can create a chemoattractant gradient spontaneously, and consequently move outwards. In Fig. S8(d), we can observe that at time T = 2240s, the succinate inside the swarm ring is nearly exhausted, and the bacteria swarm ring carrying information covers all the network nodes in the 3D space. Note that bacteria division affects the formation of the swarm ring only weakly; this is because the division process has a much larger time scale (in the order of hours).

Table 2. Comparing TAMSD and EMSD for different densities of cases 1 for simulated bacterium trajectory

Case 1	Time lag	TAMSD	EMSD	Error
$8x10^2$ bacteria/cm^3, with chemoattractant	10 (s)	16529	13261	% 24
$8x10^2$ bacteria/cm^3, with chemoattractant	100 (s)	736230	719880	% 2
$8x10^2$ bacteria/cm^3, with chemoattractant	400 (s)	3779700	3499200	% 8
$8x10^2$ bacteria/cm^3, with chemoattractant	800 (s)	8796900	7886600	% 11
$8x10^3$ bacteria/cm^3, with chemoattractant	10 (s)	15787	19082	% 20
$8x10^3$ bacteria/cm^3, with chemoattractant	100 (s)	741230	664580	% 11
$8x10^3$ bacteria/cm^3, with chemoattractant	400 (s)	2420200	3220700	% 33
$8x10^3$ bacteria/cm^3, with chemoattractant	800 (s)	11180000	6648500	% 40
$8x10^4$ bacteria/cm^3, with chemoattractant	10 (s)	15631	12961	% 20
$8x10^4$ bacteria/cm^3, with chemoattractant	100 (s)	597950	687400	% 14
$8x10^4$ bacteria/cm^3, with chemoattractant	400 (s)	3706100	3395200	% 9
$8x10^4$ bacteria/cm^3, with chemoattractant	800 (s)	3979800	5281200	% 32

Table 3. Slope of line fitted to TAMSD plot of a single bacterium (related to Fig. 2d in paper)

Bacteria density	Chemoattractant condition	$\alpha(\pm e)$	Diffusion type
$8x10^2$ (bacteria/cm^3)	Without chemoattractant	$1.402(\pm 0.026)$	*superdiffusion*
$8x10^2$ (bacteria/cm^3)	With chemoattractant	$1.483(\pm 0.022)$	*superdiffusion*
$8x10^3$ (bacteria/cm^3)	Without chemoattractant	$1.229(\pm 0.015)$	*superdiffusion*
$8x10^3$ (bacteria/cm^3)	With chemoattractant	$1.220(\pm 0.022)$	*superdiffusion*
$8x10^5$ (bacteria/cm^3)	Without chemoattractant	$0.79(\pm 0.020)$	*subdiffusion*
$8x10^5$ (bacteria/cm^3)	With chemoattractant	$1.07(\pm 0.010)$	*superdiffusion*
$8x10^6$ (bacteria/cm^3)	Without chemoattractant	$0.040(\pm 0.010)$	*subdiffusion*
$8x10^6$ (bacteria/cm^3)	With chemoattractant	$0.050(\pm 0.010)$	*subdiffusion*

Table 4. Goodness-of-fit for Gaussian and stable type distributions for distance a bacterium travels in lag time τ

τ	Gaussian distribution fit parameters		Goodness-of-fit for Gaussian distribution					Stable type distribution fit parameters	Goodness-of-fit for stable type distribution		
	μ	σ	Lillie test		Kolmogorov-Smirnov test						
			h	p-value	h	p-value	Test statistic	α	h	p-value	Test statistic
100	806.66	231.28	1	$1x10^{-3}$	1	$1.7578x10^{-4}$	0.0681	1.8	0	0.1944	0.0480
500	$1.69x10^3$	658.65	1	$1.5x10^{-3}$	1	0.0361	0.0630	1.9	0	0.9672	0.0220
1000	$2.14x10^3$	1001.3	1	$1x10^{-3}$	1	$5.6183x10^{-5}$	0.0721	1.8	0	0.0725	0.0600

Note 11. Dickey-Fuller test results to check bacterium motion is nonstationary

We performed a Dickey-Fuller test on the time trajectory of simulated bacteria motion in the case of 8×10^2 bacteria/cm^3 swarm density without chemoattractant (the same case analyzed and presented in Fig. 3a in the main manuscript). We also performed the same test on white noise as an example of a stationary process and on a random walk as an example of a nonstationary process to be able to compare the results. Table 5 presents the results of the Dickey-Fuller test, which checks for the existence of a unit root in time series. The time series has a unit root if 1 is a root of the process's characteristic equation. If the time series has a unit root, we can conclude that it is nonstationary. In this table, $h = 0$ rejects that the time series is nonstationary, and ADFpval presents the probability that the time series has a unit root. Table 5 shows that based on our expectation, the test rejects that the white noise time series is nonstationary, but it does not reject it in the rest of the cases.

Table 5. Dickey-Fuller test results

Case of study	Dickey-Fuller test		
	h	ADFpval	ADFstat
White noise	0	0	- 95.4111
Random Walk	1	0.29966	- 0.9712
8×10^2 bacteria/cm^3, without chemoattractant, bacterium motion in 3 dimensions space	1	0.8632	0.6863
1×10^4 bacteria/cm^3, without chemoattractant, bacterium motion in 3 dimensions space	1	0.8807	0.7786
1×10^5 bacteria/cm^3, without chemoattractant, bacterium motion in 3 dimensions space	1	0.7781	0.3227

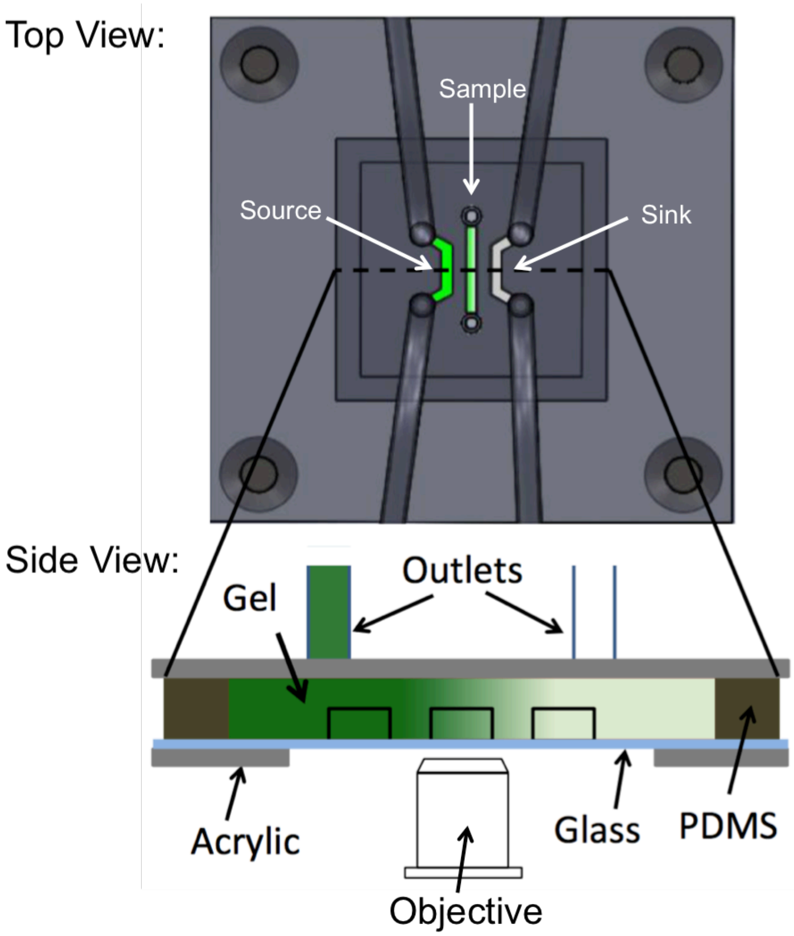

Figure S9. Experimental Setup. The top and side view of the three-channel microfluidic concentration gradient generator used in the experimental study is shown. The device consists of three parallel microfluidic channels: source channel (left) containing a high concentration of the chemoattractant (indicated by the green color), sink channel (right) containing a low concentration of the chemoattractant, and the sample channel (center) containing the bacterial cells. An agarose gel, through which molecules can diffuse, separates the channels. A linear concentration gradient develops in the sample channel at steady state.

Note 12. Tracking the bacteria in experiments

To connect bacteria positions in individual frames and form the time trajectories of bacteria up to tens of seconds in length, we used a nearest neighbor algorithm. In other words, we connected the disjoint path segments by using a weighting function, which determined the likelihood that two path segments were produced serially by the same bacteria. Equation (32) explains this weighting function in more detail. This algorithm helps to connect positions of bacteria in disjoint path segments which were produced serially by the same bacteria in different frames.

$$w = c_2 d^2 + c_3(\Delta v)^2 + c_4|\Delta\theta| + c_5\Delta t \tag{32}$$

In equation (32), Δv is the change in speed between the end of the first trajectory (previous path segment) and the start of the second trajectory (next path segment). The speed was calculated from trajectories smoothed with a five frame moving average. $\Delta\theta$ is the change in heading during a tumble. The heading was defined as the direction of the instantaneous velocity, which was calculated from the smoothed trajectories. Δt is the change in time. d is the difference in position between the start of the second trajectory and where as object moving at the final speed and heading of the first trajectory would be at a time Δt after the end of that trajectory. The coefficients c_i are empirically fitted constants. Further details about the tracking algorithm are described in [41].

References

[1] Mardia KV. 1974 Applications of some measures of multivariate skewness and kurtosis in testing normality and robustness studies. *Sankhyā: The Indian Journal of Statistics, Series B*: 115-128.

[2] Mardia KV. 1970 Measures of multivariate skewness and kurtosis with applications. *Biometrika* 57: 519-530.

[3] Mardia KV, Zemroch PJ. 1975 Algorithm AS 84: Measures of multivariate skewness and kurtosis. *Applied Statistics*: 262-265.

[4] Doornik JA, Hansen H. 1994 An omnibus test for univariate and multivariate normality. Discussion Paper W4&91. Nuffield College, Oxford, UK.

[5] D'Agostino RB. 1970 Transformation to normality of the null distribution of g1. Biometrika 57: 679-681.

[6] Shenton LR, Bowman KO. 1977 A bivariate model for the distribution of√ b1 and b2. *Journal of the American Statistical Association* 72: 206-211.

[7] Henze N, Zirkler B. 1990 A class of invariant consistent tests for multivariate normality. *Communications in Statistics-Theory and Methods* 19: 3595-3617.

[8] Henze N, Wagner T. 1997 A new approach to the BHEP tests for multivariate normality. *Journal of Multivariate Analysis* 62: 1-23.

[9] Johnson RA, Wichern DW. 2002 *Applied Multivariate Statistical Analysis* Vol. 5. Upper Saddle River, NJ: Prentice hall.

[10] Mecklin CJ, Mundfrom DJ. 2003 On using asymptotic critical values in testing for multivariate normality. *InterStat* 1.

[11] Johnson RA, Wichern DW. 1992 Applied Multivariate Statistical Analysis. 3rd. ed. New-Jersey:Prentice Hall.

[12] Mecklin CJ, Mundfrom DJ. 2005 A Monte Carlo comparison of the Type I and Type II error rates of tests of multivariate normality. *Journal of Statistical Computation and Simulation* 75: 93-107.

[13] Royston JP. 1982 An extension of Shapiro and Wilk's W test for normality to large samples. *Applied Statistics* 2: 115-124.

[14] Royston JP. 1983 Some techniques for assessing multivarate normality based on the Shapiro-Wilk W. *Applied Statistics* 32: 121-133.

[15] Royston P. 1992 Approximating the Shapiro-Wilk W-Test for non-normality. *Statistics and Computing* 2: 117-119.

[16] Royston P. 1995 Remark AS R94: A remark on algorithm AS 181: The W-test for normality. *Applied Statistics* 44, 547-551.

[17] Shapiro SS, Wilk MB. *An analysis of variance test for normality (complete samples)* (Doctoral dissertation, Rutgers, The State University).

[18] Ihlen EA. 2012 Introduction to multifractal detrended fluctuation analysis in Matlab. *Frontiers in physiology* 3.

[19] Barkai N, Leibler S. 1997 Robustness in simple biochemical networks. *Nature* 387.6636: 913-917.

[20] Wei G, Bogdan P, Marculescu R. 2013 Efficient modeling and simulation of bacteria-based nanonetworks with BNSim. *Selected Areas in Communications, IEEE Journal on* 31: 868-878.

[21] Sneddon MW, Faeder JR, Emonet T. 2010 Efficient modeling, simulation and coarse-graining of biological complexity with NFSim. *Nature Methods* 8: 177–183.

[22] Kalinin YV, Jiang L, Tu Y, Wu M. 2009 Logarithmic sensing in Escherichia coli bacterial chemotaxis. *Biophysical journal* 96: 2439-2448.

[23] Tu Y. 2013 Quantitative modeling of bacterial chemotaxis: Signal amplification and accurate adaptation. *Annual review of biophysics* 42: 337.

[24] Kalinin YV, et al. 2009 Logarithmic sensing in escherichia coli bacterial chemotaxis. *Biophysical journal* 96: 2439-2448.

[25] Vladimirov N, Lovdok L, Lebiedz D, Sourjik V. 2008 Dependence of bacterial chemotaxis on gradient shape and adaptation rate. *PLoS computational biology* 4, e1000242.

[26] Sneddon MW, Pontius W, Emonet T. 2012 Stochastic coordination of multiple actuators reduces latency and improves chemotactic response in bacteria. *Proceedings of the National Academy of Sciences* 109.3: 805-810.
[27] Duke TAJ, Novere NL, Bray D. 2001 Conformational spread in a ring of proteins: a stochastic approach to allostery. *Journal of molecular biology* 308.3: 541-553.
[28] Tu Y, Shimizu ThS, Berg HC. 2008 Modeling the chemotactic response of Escherichia coli to time-varying stimuli. *Proceedings of the National Academy of Sciences* 105.39: 14855-14860.
[29] Vaknin A, Berg HC. 2007 Physical responses of bacterial chemoreceptors. *Journal of molecular biology* 366.5: 1416-1423.
[30] Barkai N, Leibler S. 1997 Robustness in simple biochemical networks. *Nature* 387.6636: 913-917.
[31] Cheng S, et al. 2007 A hydrogel-based microfluidic device for the studies of directed cell migration. *Lab on a Chip* 7.6: 763-769.
[32] Keller EF, Segel LA. 1971 Model for chemotaxis. *Journal of Theoretical Biology* 30.2: 225-234.
[33] Chen KC, Ford RM, Cummings PT. 1998 Mathematical models for motile bacterial transport in cylindrical tubes. *Journal of theoretical biology* 195.4: 481-504.
[34] Portillo IG, Campos D, Méndez V. 2011 Intermittent random walks: transport regimes and implications on search strategies. *Journal of Statistical Mechanics: Theory and Experiment* 2011.02.
[35] Thiel F, Schimansky-Geier L, Sokolov IM. 2012 Anomalous diffusion in run-and-tumble motion. *Physical Review E* 86.2.
[36] Ahmed T, Stocker R. 2008 Experimental verification of the behavioral foundation of bacterial transport parameters using microfluidics. *Biophysical journal* 95.9: 4481-4493.
[37] Ahmed T, Shimizu TS, Stocker R. 2010 Bacterial chemotaxis in linear and nonlinear steady microfluidic gradients. *Nano letters* 10.9: 3379-3385.
[38] Park H, et al. 2011 Fine-tuning of chemotactic response in E. coli determined by high-throughput capillary assay. *Current microbiology* 62.3: 764-769.
[39] Cluzel P, Surette M, Leibler S. 2000 An ultrasensitive bacterial motor revealed by monitoring signaling proteins in single cells. *Science* 287.5458: 1652-1655.
[40] Budrene EO, Berg HC. 1995 Dynamics of formation of symmetrical patterns by chemotactic bacteria, *Nature* 376: 49–53.
[41] Edwards MR, Carlsen RW, Zhuang J, Sitti M. 2014 Swimming characterization of *Serratia marcescens* for bio-hybrid micro-robotics. *J. Micro-Bio Robotics* 9: 47-60.
[42] Berg HC, Brown DA. 1972 Chemotaxis in *Escherichia coli* analysed by three-dimensional tracking. *Nature* 239: 500–504.
[43] Demir M, Dourche C, Yoney A, Libchaber A, Salman H. 2011 Effects of population density and chemical environment on the behavior of *Escherichia coli* in shallow temperature gradients. *Phys Bio* 8.
[44] Zhuang J, et al. 2014 Analytical Modeling and experimental characterization of chemotaxis in *Serratia marcescens*. *Phys. Rev.* 89.
[45] Zhuang J, Wei G, Carlsen RW, Edwards MR, Marculescu R, Bogdan P, Sitti M. 2014 Analytical modeling and experimental characterization of chemotaxis in *Serratia marcescens*. *Phys Rev* 89.
[46] Budrene EO, Berg H. 1991 Complex patterns formed by motile cells of escherichia coli, *Nature* 349: 630-633.
[47] Sengupta A, Teeffelen SV, Löwen H. 2009 Dynamics of a microor- ganism moving by chemotaxis in its own secretion, *Physical Rev. E* 80.
[48] Inoue M, Kaneko K. 2008 Conditions for self-consistent aggregation by chemotactic particles, *Physical Rev.* 77.
[49] Guzmán-Vargas L, Angulo-Brown F. 2003 Simple model of the aging effect in heart interbeat time series. *Physical Review* 67.5.
[50] Burov S, Jeon JH, Metzler R, Barkai E. 2011 Single particle tracking in systems showing anomalous diffusion: the role of weak ergodicity breaking. *Physical Chemistry Chemical Physics* 13: 1800-1812.
[51] Korobkova E, Emonet T, Vilar J, Shimizu T, Cluzel P. 2004 From molecular noise to behavioural variability in a single bacterium, *Nature* 428: 574–578.

[52] Wei G, Bogdan P, Marculescu R. 2013 Bumpy rides: Modeling the dynamics of chemotactic interacting bacteria. *Selected Areas in Communications, IEEE Journal* 31.12: 879-890.
[53] Tsallis C, et al. 1995 Statistical-mechanical foundation of the ubiquity of Lévy distributions in nature. *Physical Review Letters* 75.20.
[54] Ihlen EA. 2012 Introduction to multifractal detrended fluctuation analysis in Matlab. *Frontiers in physiology* 3.

[55] Kantelhardt JW, Zschiegner SA, Koschielny-unde E, Havlin S, Bunde A, Stanley HE. 2002 Multifractal detrended fluctuation analysis of nonstationary time series. *Physica A: Statistical Mechanics and its Applications* 316:87-114. (doi: 10.1016/S0378-4371(02)01383-3)
